# Targeted deep sequencing improves outcome stratification in chronic myelomonocytic leukemia with low risk cytogenetic features

**DOI:** 10.18632/oncotarget.10937

**Published:** 2016-07-29

**Authors:** Laura Palomo, Olga Garcia, Montse Arnan, Blanca Xicoy, Francisco Fuster, Marta Cabezón, Rosa Coll, Vera Ademà, Javier Grau, Maria-José Jiménez, Helena Pomares, Sílvia Marcé, Mar Mallo, Fuensanta Millá, Esther Alonso, Anna Sureda, David Gallardo, Evarist Feliu, Josep-Maria Ribera, Francesc Solé, Lurdes Zamora

**Affiliations:** ^1^ MDS Research Group, Josep Carreras Leukaemia Research Institute, ICO-Hospital Germans Trias i Pujol, Universitat Autònoma de Barcelona, Badalona, Spain; ^2^ Departament de Bioquímica i Biologia Molecular, Universitat Autònoma de Barcelona, Badalona, Spain; ^3^ Hematology Service, ICO-Hospital Germans Trias i Pujol, Josep Carreras Leukaemia Research Institute, Universitat Autònoma de Barcelona, Badalona, Spain; ^4^ Hematology Service, ICO-Hospital Duran i Reynals, Barcelona, Spain; ^5^ Hematology Service, ICO-Hospital Josep Trueta, Girona, Spain

**Keywords:** chronic myelomonocytic leukemia, normal karyotype, gene mutations, targeted deep sequencing, prognostic factors

## Abstract

Clonal cytogenetic abnormalities are found in 20-30% of patients with chronic myelomonocytic leukemia (CMML), while gene mutations are present in >90% of cases. Patients with low risk cytogenetic features account for 80% of CMML cases and often fall into the low risk categories of CMML prognostic scoring systems, but the outcome differs considerably among them. We performed targeted deep sequencing of 83 myeloid-related genes in 56 CMML patients with low risk cytogenetic features or uninformative conventional cytogenetics (CC) at diagnosis, with the aim to identify the genetic characteristics of patients with a more aggressive disease. Targeted sequencing was also performed in a subset of these patients at time of acute myeloid leukemia (AML) transformation. Overall, 98% of patients harbored at least one mutation. Mutations in cell signaling genes were acquired at time of AML progression. Mutations in *ASXL1*, *EZH2* and *NRAS* correlated with higher risk features and shorter overall survival (OS) and progression free survival (PFS). Patients with *SRSF2* mutations associated with poorer OS, while absence of *TET2* mutations (*TET2*wt) was predictive of shorter PFS. A decrease in OS and PFS was observed as the number of adverse risk gene mutations (*ASXL1*, *EZH2, NRAS* and *SRSF2*) increased. On multivariate analyses, CMML-specific scoring system (CPSS) and presence of adverse risk gene mutations remained significant for OS, while CPSS and TET2wt were predictive of PFS. These results confirm that mutation analysis can add prognostic value to patients with CMML and low risk cytogenetic features or uninformative CC.

## INTRODUCTION

Chronic myelomonocytic leukemia (CMML) is a hematopoietic stem cell disorder with features from both myelodysplastic syndromes (MDS) and myeloproliferative neoplasms (MPN) [1]. The original French-American-British (FAB) criteria identifies two variants based on leukocyte count (myelodysplastic [<13×10^9^/L], MD-CMML, and myeloproliferative [>13×10^9^/L], MP-CMML), while the 2008 World Health Organization (WHO) classification distinguishes two categories (CMML-1 and CMML-2) according to blast percentage in bone marrow (BM) or peripheral blood (PB) [1, 2]. Very recently, the 2016 revision of the WHO criteria has proposed the inclusion of a third CMML subtype, CMML-0, based on recent findings that demonstrate that these three CMML subtypes have different clinical outcomes [3, 4].

Clonal cytogenetic abnormalities are not frequent in CMML (20-30%), whereas gene mutations have been reported in >90% of patients at diagnosis [5–7]. Prognostic impact of cytogenetic alterations in CMML was first explored by the Spanish MDS group and recently reviewed by the Mayo Clinic-French Consortium [5, 8]. According to both studies, up to 80% of CMML patients present with low risk cytogenetic features (normal karyotype, isolated -Y or sole der(3q)).

During the past years, several studies have reported recurrent gene mutations in CMML, being mutations in *TET2* (50-60%), *ASXL1* (40-50%) and *SRSF2* (40-50%) the most frequent [7, 9, 10]. Less frequent mutations (10-30%) have also been described in *RUNX1, CBL, K/NRAS, EZH2, UTX, DNMT3A* and *JAK2* genes [6, 7, 10–12]. Prognostic relevance of mutations in *ASXL1*, *TET2*, *RUNX1*, *CBL* and *NRAS* has been demonstrated on univariate survival analyses on CMML [7, 13, 14], but only *ASXL1* mutations seem to retain this impact on multivariate models [15, 16].

Several prognostic scoring systems have been proposed for CMML in the past years. The CMML-specific scoring system (CPSS) was developed by the Spanish MDS group and includes CMML-2, MP-CMML, transfusion dependency and cytogenetic risk stratification as independent adverse prognostic factors [17]. Other novel CMML-specific scoring systems, like the Groupe Francophone des Myélodysplasies (GFM) CMML model [15] and the Molecular Mayo model [16], include similar biological parameters but exclude cytogenetic abnormalities. These two models introduce for the first time the use of molecular criteria, such as the presence of mutations in *ASXL1*.

A significant subset of CMML patients fall into the low risk cytogenetic category and most of them present with normal karyotype, but the median overall survival (OS) and the risk of AML progression differ considerably among them [17]. With the aim to identify a subgroup of patients with a more aggressive disease, we performed targeted deep sequencing in 56 patients with CMML and low risk cytogenetic features or no metaphases and explored the prognostic value of gene mutations.

## RESULTS

### Characteristics of CMML patients

A total of 56 patients with CMML and low risk cytogenetic features or uninformative conventional cytogenetics (CC) were included in the study. Median follow-up of alive patients was 36 months (range, 5.8 to 83.5 months). Main clinical and biological characteristics of patients are summarized in Table 1. Median age at diagnosis was 72 years and the series included 37 (66%) males and 19 (34%) females. Following the FAB criteria [2], 46 (82%) patients were classified as MD-CMML and 10 (18%) as MP-CMML, while according to the 2008 WHO classification [1], 49 (87%) cases corresponded to CMML-1 and 7 (13%) to CMML-2. Progression to AML was observed in 16 (29%) patients. Risk stratification of patients was based on the CPSS [17] and the GFM CMML model [15] (Table 1).

**Table 1:** Main clinical and hematological characteristics of CMML patients at diagnosis (n=56)

Variable	Median (range)	N (%)
**Age, years**	72 (48-89)	22/56 (39)
<70		34/56 (61)
≥70		
**Gender**		37/56 (66)
Male		19/56 (34)
Female		
**FAB classification**		46/56 (82)
Myelodysplastic (CMML-MD)		10/56 (18)
Myeloproliferative (CMML-MP)		
**WHO classification**		49/56 (87)
CMML-1		7/56 (13)
CMML-2		
**Hemoglobin level, g/dL**	12.1 (7.2-16.1)	8/56 (14)
<10		48/56 (86)
≥10		
**Leukocyte count, x10^9^/L**	8.0 (3.2-45.0)	46/56 (82)
<13		10/56 (18)
≥13		
**Platelet count, x10^9^/L**	139.0 (25.0-481.0)	18/56 (32)
<100		38/56 (68)
≥100		
**Neutrophil count, x10^9^/L**	4.1 (0.8-30.2)	9/56 (16)
<1.8		47/56 (84)
≥1.8		
**Blasts in BM, %**	2.0 (0.0-15.0)	53/56 (95)
<10		3/56 (5)
≥10		
**RBC transfusion dependency**		50/56 (89)
No		6/56 (11)
Yes		
**Splenomegaly**		34/43 (79)
No		9/43 (21)
Yes		
**Cytogenetics**		51/56 (91)
Normal karyotype		3/56 (5)
Isolated -Y		2/56 (4)
Uninformative CC		
**CPSS risk group** [17]		42/56 (75)
** Low**		9/56 (16)
Intermediate-1		5/56 (9)
Intermediate-2		
**GFM CMML model** [15]		37/56 (66)
** Low**		17/56 (30)
Intermediate		2/56 (4)
High		
**Progression to AML**		40/56 (71)
No		16/56 (29)
Yes		

BM: bone marrow; RBC: red blood cell; Uninformative CC: cases with no metaphases; AML: acute myeloid leukemia

### Conventional cytogenetics

Conventional cytogenetics was performed in all patients at diagnosis (n=56) and in 12 patients at the time of AML transformation. All patients had low risk cytogenetic features at diagnosis (51 with normal karyotype and three with isolated -Y) except for two cases in which no metaphases were obtained, therefore being considered as uninformative for CC (Table 1). At the time of AML progression, 6 (50%) patients still presented with normal karyotype, while the other 6 (50%) cases had acquired chromosomal aberrations. In 4 out of these 6 patients, these corresponded to high risk cytogenetic abnormalities according to CPSS [5].

### Targeted deep sequencing

Targeted deep sequencing was performed in a total of 64 samples, with a mean depth per base per sample of 1256-fold (1256x). More than 95% of the target sequences were analyzed with >100 independent reads and >99% with at least 30 reads. After excluding sequencing and mapping errors a mean of 299 single nucleotide variants (SNVs) and insertions/deletions (indels) were called per sample. After filtering non-silent variants and excluding known polymorphisms, a mean of 4 variants per sample were called as high-probability somatic changes.

### Spectrum of gene mutations at diagnosis

**Figure 1:**
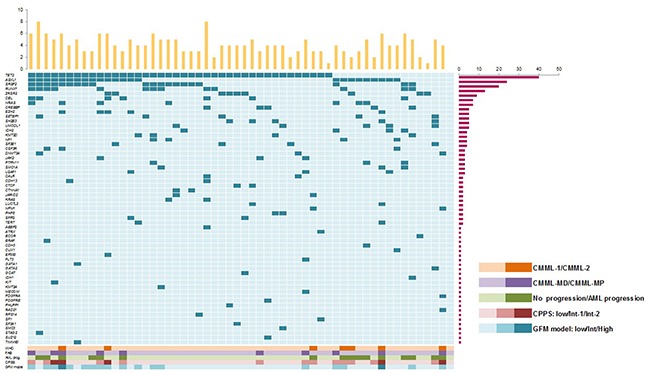
Distribution of the affected genes across the 56 CMML patients at diagnosis One gene is represented in each line and one patient in each column. Bars at the right represent the number of mutations present in each gene, while columns at the top represent the number of mutations per patient. At the bottom correlations of mutations with WHO, FAB, AML progression, CPSS and CFM model.

Across the entire cohort, 98% (55/56) of patients harbored at least one mutation. Details of all the variants detected in the series can be seen in Supplementary Table S1. Overall, 2 (4%) patients had 1 mutation, 5 (9%) had 2 concurrent mutations, 12 (21%) had 3, 15 (27%) had 4, 11 (20%) had 5, 8 (14%) had 6, and 2 (4%) had 8 (Supplementary Figure S1A). Distribution of the detected mutations across the CMML patients are described in Figure 1. Most frequently affected genes (in >10% of patients) were *TET2* (71%), *ASXL1* (43%) and *SRSF2* (36%); followed by *RUNX1* (23%), *ZRSR2* (16%), *CBL* (13%) and *NRAS* (13%). Mutations detected in 5-10% of patients were found in the following genes: *EZH2*, *CREBBP*, *UMODL1*, *SETBP1*, *SH2B3*, *NF1*, *IDH2*, *SF3B1*, *KMT2D, CSF3R*, *JAK2, PTPN11*, *SMC1A, U2AF1* and *DNMT3A* (Supplementary Figure S1B). The list of all the affected genes can be seen in Supplementary Table S2, and the mutation type distribution according to each affected gene can be seen in Supplementary Figure S1C. Most of these genes are involved in cell signaling, epigenetic mechanisms and spliceosome machinery (Supplementary Figure S1D). We then examined the correlation between gene mutations in order to identify possible functional interactions across the different affected genes. All genes were included in all statistical analyses, but to ensure a minimum statistical accuracy, from now on we will focus on mutations detected in at least 5 patients. Mutations in *ASXL1* frequently co-occurred with mutations in *NRAS* (*P*=0.035) and *EZH2* (*P*=0.011). A positive correlation was also found between *RUNX1* and *CBL* (*P*=0.043). Finally, mutations in *SRSF2* correlated with mutations in *CBL* (*P*=0.043), but were mutually exclusive with mutations in *ZRSR2* (*P*=0.019).

### Coexistence of gene mutations and loss of heterozygosity

Most of the patients in this cohort (n=48/56, 85.7%) had been previously studied by our group using single nucleotide polymorphism arrays (SNP-A) [18]. Therefore, we investigated whether some of the mutations detected in the present study correlated with the alterations previously detected by SNP-A. Interstitial copy number neutral loss of heterozygosity (CNN-LOH) was detected in 14 of these patients, 10 of which also presented with one mutation affecting a gene located in the region with CNN-LOH. All patients (n=4) with CNN-LOH in 4q24q35 region harbored a *TET2* mutation; all patients (n=3) with CNN-LOH in 11q13.3q25 had a mutation in *CBL*; one patient with CNN-LOH in 7q22.1q36.3 showed a *EZH2* mutation, another one with CNN-LOH in 12q21.2q24.33 had a *KRAS* mutation and one patient with CNN-LOH in 17q25.3 harbored a *SRSF2* mutation (Supplementary Table S3). Interstitial CNN-LOH from the four remaining patients affected regions that did not include any of the studied genes (Supplementary Table S3).

### Acquisition of mutations during AML progression

Targeted deep sequencing was performed at time of AML transformation in seven patients and at time of CMML-2 progression in one patient. The spectrum of mutations detected per patient was different between diagnosis and AML progression for all except from one patient. In the case that evolved from CMML-1 to CMML-2 it did not differ (Table 2). Number of mutations per patient was higher at time of AML progression in 5/7 (71.4%) patients. Considering alterations detected by both CC and sequencing, median number of alterations at time of progression was higher than at diagnosis (5 alterations at progression *vs*. 3 alterations at diagnosis, *P*=0.017). Mutations acquired in all but one patient that progressed to AML affected genes involved in cell signaling pathways that affect cell division, growth, differentiation and survival; such as *BRAF, FLT3, KRAS, PTPN11* and *NRAS*. Of note, the remaining patient that progressed to AML did not acquire any additional mutation, but presented with the intermediate cytogenetic abnormality t(8;16)(p11;13), detected by CC at time of AML progression.

**Table 2:** List of affected genes in CMML patients that were studied at diagnosis and at time of AML or CMML-2 progression (n=8)

Data at diagnosis				Data at progression			
Diagnosis	N of genes	Gene	Mutation Freq. (%)	Progression	N of genes	Gene	Mutation Freq. (%)
CMML-1	5	*SETBP1*	45	CMML-2	5	*SETBP1*	49
		*UMODL1*	46			*UMODL1*	47
		*SH2B3*	43			*SH2B3*	41
		*SF3B1*	48			*SF3B1*	45
		*GATA2*	46			*GATA2*	41
CMML-1	5	*ASXL1*	49	AML	5	*ASXL1*	47
		*UMODL1*	50			*UMODL1*	51
		*CDH3*	40			*CDH3*	20
		*NRAS*	15			*NRAS*	6
		***PTPN11***	**6**				
						***BRAF***	**9**
CMML-1	2	*RUNX1*	52	AML	4	*RUNX1*	52
		*TET2*	45			*TET2*	43
						***SRSF2***	**24**
						***FLT3***	**22**
CMML-1	3	*TET2*	47	AML	6	*TET2*	48
		*CBL*	36			*CBL*	5
		*ASXL1*	43			*ASXL1*	46
						***KMT2D***	**51**
						***KRAS***	**42**
						***AEBP2***	**32**
CMML-2	3	*JARID2*	51	AML	4	*JARID2*	48
		*TET2*	44			*TET2*	43
		*NPM1*	31			*NPM1*	30
						***GNAS***	20
CMML-2	6	*ASXL1*	52	AML	6	*ASXL1*	49
		*CSF3R*	51			*CSF3R*	47
		*SRSF2*	41			*SRSF2*	12
		*TET2*	44			*TET2*	46
		*NRAS*	47			*NRAS*	47
		*EZH2*	39			*EZH2*	43
CMML-2	2	*ASXL1*	43	AML	4	*ASXL1*	48
		*ZRSR2*	19			*ZRSR2*	22
						***KMT2D***	**22**
						***PTPN11***	**9**
CMML-2	3	*ASXL1*	34	AML	4	*ASXL1*	39
		*RUNX1*	45			*RUNX1*	50
		*NF1*	6			*NF1*	8
						***NRAS***	**5**

Genes that are only affected at time of diagnosis or progression are highlighted in bold

### Correlations between gene mutations and clinical variables

We investigated the correlation between mutations detected at diagnosis and main clinical and biological parameters of the patients, including age, sex, CMML FAB and WHO subtypes, BM and PB cell counts, RBC transfusion dependency, presence of splenomegaly, CPSS and GFM models and progression to AML. Mutations in *EZH2* gene associated with WHO 2008 CMML-2 subtype (*P*=0.011), FAB CMML-MP subtype/leukocyte count (*P*=0.035) and higher risk groups according to CPSS (*P*<0.001) and GFM (*P*=0.001) models. Mutations in *NRAS* correlated with FAB CMML-MP subtype/leukocyte count (*P*=0.015), presence of splenomegaly (*P*>0.001) and age <70 years (*P*=0.012). *ASXL1* mutations associated with AML progression (*P*=0.034), age <70 years (*P*=0.015) and higher risk groups according to the CPSS (*P*=0.014) and GFM (*P*=0.001) models. *SRSF2* mutations correlated with platelet count <100 x10^9^/L and higher risk groups according to GFM model (*P*=0.025). Even though *JAK2* mutations were only present in three patients, it is worth highlighting, because it has been previously reported [19], that they associated with FAB CMML-MP subtype/leukocyte count (*P*=0.004). Interestingly, mutations in *TET2* gene were the only ones associated with good prognosis features, such as Hemoglobin>10g/dL (*P*=0.005), not progression to AML (*P*=0.008) and lower risk groups according to CPSS (*P*=0.036).

### Univariate survival analyses

**Figure 2:**
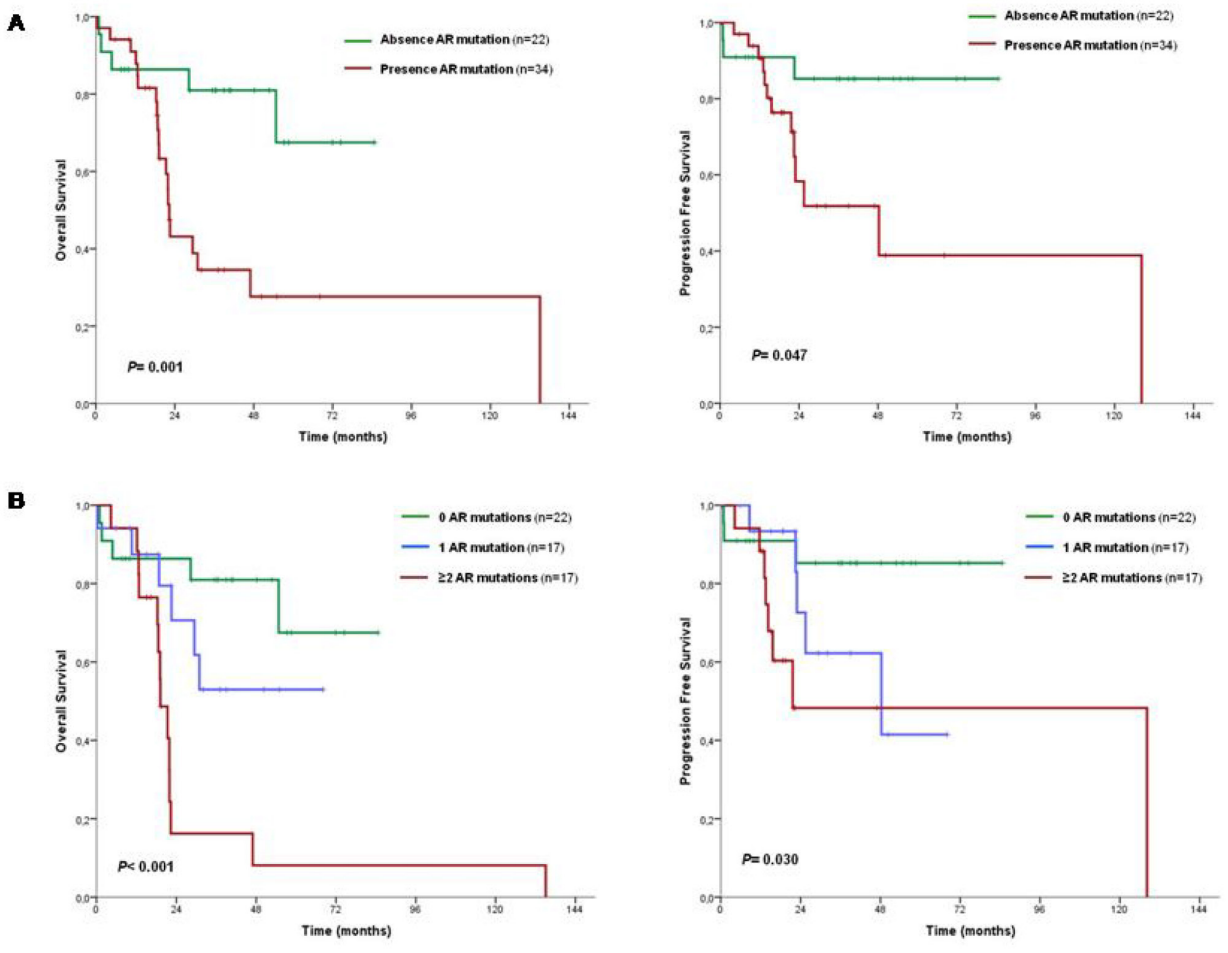
Prognostic impact of gene mutations **A.** OS and PFS curves according to presence or absence of an adverse risk gene; **B.** OS and PFS curves according to number of mutations in an adverse risk gene. See Table 3 for 3-year percentage overall survival and progression free survival and confidence intervals. AR mutations: adverse risk gene mutations (*ASXL1, EZH2, NRAS, SRSF2*).

We then explored the impact of clinical, biological and genetic data on patients’ outcome (Table 3). Median OS and progression free survival (PFS) of the cohort were 47 months (IC95% 15-79) and 128 months (NA), respectively. The following clinical and biological variables were predictive of both OS and PFS: CMML WHO subtype, CMML FAB subtype, transfusion dependency, presence of splenomegaly, hemoglobin level, leukocyte count, CPSS risk group, alternative CPSS risk group and GFM CMML model. In addition, BM blast percentage and age were also predictive of PFS. Regarding genetic features, total number of mutations was predictive of OS and PFS when patients were stratified into the following subgroups: 0-3 mutations, 4-5 mutations, >5 mutations (Table 3, Supplementary Figure S2A). Focusing on specific genes, mutations in *ASXL1, NRAS* and *EZH2* associated with both shorter OS and PFS. Furthermore, mutations in *SRSF2* only associated with inferior OS, while absence of *TET2* mutations (TET2wt) associated with inferior PFS but did not correlate with OS (Table 3, Supplementary Figure S2B). Overall, 34/56 (61%) of patients presented with at least one adverse risk gene mutation (*ASXL1, EZH2, NRAS* and *SRSF2*). Presence of a mutation in one of these genes correlated with both shorter OS and PFS (Table 3, Figure 2A). Moreover, a decrease in OS and PFS was observed as the number of adverse risk mutations increased. Patients were classified in three groups according to the number of mutations in these genes (0, 1, ≥2), which associated with poorer OS and PFS (Table 3, Figure 2B). Even when patients were classified in four groups (0, 1, 2, ≥3), the statistical association was maintained (Table 3, Supplementary Figure S2C). Recently, Patnaik *et al*. reported a prognostic interaction between *ASXL1* and *TET2* mutations in CMML [6], confirming the negative impact in OS imparted by *ASXL1* mutations and suggesting a favorable impact from *TET2* mutations in the absence of *ASXL1* mutations. We investigated this interaction in our cohort of patients and observed that the different combinations between *ASXL1* and *TET2* mutations were able to stratify patients in subgroups with significantly different OS (Table 3, Supplementary Figure S2D). Regarding PFS, patients with mutations only in *TET2* presented a better outcome, but we observed a high overlap between the rest of the categories (Table 3, Supplementary Figure S2D). In order to delineate the benefit of *TET2* mutations related to other adverse mutations, we investigated the prognostic interaction between *TET2* mutations and adverse risk genes excluding *ASXL1*, which was able to separate the patients in four distinct prognostic groups with clear different OS and PFS (Table 3, Supplementary Figure S2E). Overall, focusing on patients with mutations in one of these genes (*EZH2*, *NRAS* or *SRSF2*), regardless of *TET2* status, they had an unfavorable prognosis, even though patients with *TET2* mutations showed a better prognosis in this subset. On the other hand, in the absence of adverse risk gene mutations, patients with *TET2* mutations again had a better outcome than patients without, suggesting a protective role for *TET2* mutations.

**Table 3:** Overall survival analyses and progression free survival according to the main clinical, hematological and genetic characteristics of CMML patients at diagnosis (n=56)

Variable	Overall survival (OS)		Progression free survival (PFS)	
	3 year % OS (95% CI)	*Log-rank P* value	3 year % PFS (95% CI)	*Log-rank P* value
WHO classification				
CMML-1	57 (41, 73)	**0.020**	75 (60, 90)	**<0.001**
CMML-2	29 (0, 63)		19 (0, 52)	
FAB classification				
MD-CMML	61 (45, 77)	**0.007**	74 (59, 89)	**0.036**
MP-CMML	23 (5, 51)		38 (1, 75)	
Sex				
Male	50 (32, 68)	0.246	68 (50, 86)	0.916
Female	61 (34, 84)		68 (44, 92)	
Age (years)				
<70 years	37 (14, 60)	0.322	42 (18, 67)	**0.003**
≥70 years	64 (47, 81)		85 (71, 99)	
Hemoglobin level				
<10 g/dL	17 (0, 46)	**0.045**	42 (2, 82)	**0.009**
≥10 g/dL	60 (45, 75)		72 (57, 87)	
Leukocyte count				
<13×10^9^/L	61 (45, 77)	**0.007**	74 (59, 89)	**0.036**
≥13×10^9^/L	23 (5, 51)		38 (1, 75)	
Platelet count				
<100×10^9^/L	53 (27, 79)	0.797	58 (31, 85)	0.302
≥100×10^9^/L	54 (37, 71)		72 (55, 89)	
Neutrophil count				
<1,8×10^9^/L	65 (33, 97)	0.880	73 (41, 100)	0.956
≥1,8×10^9^/L	52 (36, 58)		66 (50, 82)	
Blasts in BM				
<5%	58 (42, 74)	0.499	76 (61, 91)	**0.008**
≥5%	40 (10, 70)		40 (10, 70)	
Splenomegaly				
Absent	68 (52, 84)	**0.001**	78 (62, 94)	**0.004**
Present	0 (NA)		0 (NA)	
Transfusion requirement				
Independent	65 (50, 80)	**0.001**	74 (59, 89)	**0.010**
Dependent	0 (NA)		0 (NA)	
CPSS				
Low	63 (47, 79)	**<0.001**	81 (67, 95)	**<0.001**
Intermediate-1	50 (15, 85)		39 (5, 73)	
Intermediate-2	0 (NA)		0 (NA)	
Alternative CPSS				
Low	70 (53, 87)	**0.005**	82 (67, 97)	**<0.001**
Intermediate-1	26 (2, 50)		39 (6, 72)	
Intermediate-2	25 (0, 58)		25 (0, 68)	
GFM CMML model	67 (50, 84)	**<0.001**	79 (64, 94)	**<0.001**
Low	31 (6, 56)		42 (8, 76)	
Intermediate	0 (NA)		0 (NA)	
Number of mutations				
0-3	70 (48, 92)	**<0.001**	75 (53, 97)	**0.001**
4-5	61 (39, 83)		80 (61, 99)	
>5	10 (0, 29)		15 (0, 42)	
*ASXL1*				
Wild-type	69 (52, 86)	**0.027**	81 (66, 96)	**0.015**
Mutated	30 (2, 52)		45 (13, 77)	
*NRAS*				
Wild-type	62 (47, 77)	**<0.001**	74 (59, 89)	**0.005**
Mutated	0 (NA)		0 (NA)	
*EZH2*				
Wild-type	58 (43, 73)	**0.002**	72 (57, 87)	**0.004**
Mutated	20 (0, 55)		27 (0, 71)	
*SRSF2*				
Wild-type	64 (47, 81)	**0.049**	69 (52, 86)	0.835
Mutated	35 (12, 58)		65 (37, 93)	
*TET2*				
Wild-type	41 (14, 68)	0.476	41 (14, 68)	**0.005**
Mutated	59 (42, 76)		80 (65, 95)	
Presence of adverse risk gene mutations^a^				
No	81 (64, 98)	**0.008**	85 (69, 100)	**0.031**
Yes	35 (16, 54)		52 (29, 75)	
Number of adverse risk gene mutations^a^				
0	81 (64, 98)	**<0.001**	85 (69, 100)	**0.030**
1	53 (25, 81)		16 (0, 36)	
≥2	62 (32, 92)		48 (19, 77)	
Number of adverse risk gene mutations^a^				
0	81 (64, 98)	**0.001**	85 (69, 100)	**0.047**
1	53 (25, 81)		62 (32, 92)	
2	20 (0, 44)		54 (22, 86)	
≥3	0 (NA)		0 (NA)	
Combination of *TET2* and *ASXL1* mutations				
*TET2*mut-*ASXL1*wt	78 (61, 95)	**0.001**	96 (16, 100)	**0.004**
*TET2*wt-*ASXL1*mut	47 (10, 84)		47 (10, 84)	
*TET2*wt-*ASXL1*wt	34 (0, 72)		36 (0, 75)	
*TET2*mut-*ASXL1*mut	20 (0, 44)		42 (3, 81)	
Combination of *TET2* and AR gene mutations (excluding *ASXL1*)				
*TET2*mut-*AR*wt	83 (65, 100)	**0.001**	88 (72, 100)	**<0.001**
*TET2*wt-*AR*wt	64 (31, 97)		64 (31, 97)	
*TET2*mut-ARmut	35 (12, 58)		76 (55, 97)	
*TET2*wt-*AR*mut	0 (NA)		0 (NA)	

a *ASXL1*, *EZH2*, *NRAS, SRSF2;* BM: bone marrow

CI: confidence interval; NA: Not applicable; AR genes (excluding *ASXL1*): adverse risk genes *EZH2*, *NRAS* and *SRSF2*. Variables with significant impact (*P*<0.05) in overall survival or progression free survival are highlighted in bold.

### Multivariate survival analyses

Finally, we performed an adjusted multivariate analysis including clinical, biological and genetic features that were statistically significant in the univariate analyses (Table 4). For OS, the variables that remained significant in the multivariate model, taking as baseline the low risk group, were the CPSS scoring system and the presence of at least one adverse risk gene mutation (*ASXL1, EZH2, NRAS, SRSF2*). Regarding PFS multivariate analysis, the following variables remained significant in the model: CPSS scoring system and the absence of a *TET2* mutation (*TET2*wt).

**Table 4:** Multivariate model including clinical, biological and genetic characteristics of CMML patients at diagnosis (n=56)

Overall survival				Progression free survival			
Variable	HR	95% CI	*P*	Variable	HR	95% CI	*P*
CPSS^a^:	1.2	0.4 to 3.4	**0.005**	CPSS^a^:	2.7	0.8 to 8.7	**0.002**
Int-1	6.2	2.0 to 18.8	0.695	Int-1	16.5	3.4 to 79.4	0.093
Int-2			0.001	Int-2			<0.001
Presence of adverse risk gene mutations^b,c^	2.9	1.0 to 8.2	**0.042**	*TET2* wt^d^	4.1	1.3 to 12.8	**0.013**

a Reference category: Low risk

b *ASXL1*, *EZH2*, *NRAS*, *SRSF2*

c Reference category: No mutations

d Reference category: *TET2* mutation

CI: confidence interval

## DISCUSSION

Over the past few years, next-generation sequencing (NGS) has led to a revolution in the study of hematological malignancies, with remarkable efforts to characterize the genetic basis of these disorders. In the field of CMML recent studies have reported mutations in >90% of patients, affecting genes mainly involved in the following mechanisms: epigenetic regulation (*TET2, ASXL1, EZH2, DNMT3A, IDH1/2*), spliceosome machinery (*SRSF2, ZRSR2, SF3B1, U2AF1*), cell signaling and transcription factor regulation (*NRAS, KRAS, CBL, JAK2, RUNX1*) [6, 7, 11, 15, 20, 21]. Mutations in *ASXL1, SRSF2, CBL, IDH2, EZH2*, *DNMT3A, NRAS* and *RUNX1* have been associated, in some of these studies, with poorer OS or increased risk of AML progression [6, 7, 11, 14, 15, 21–24]. However, up to date, the only gene that has shown to correlate with worse outcome on multivariate models is *ASXL1* [15, 16].

Cytogenetic abnormalities are not common in CMML (20-30%), but when present they confer a significant adverse outcome, except for isolated -Y [5, 8]. Patients with low risk cytogenetic features (normal karyotype and isolated -Y) account for approximately 80% of CMML cases and often fall into the low risk categories of CMML prognostic scores, but the OS and risk of AML transformation differs considerably among them [17]. Therefore, we have focused our study on 56 CMML patients with low risk cytogenetic abnormalities or no metaphases, since CC does not provide prognostic information in all these cases. Our aim is to identify, in this cytogenetically homogeneous cohort, the genetic characteristics of the subset of patients that present with a more aggressive disease.

By performing targeted deep sequencing using a panel of 83 myeloid-related genes, we have detected mutations in 98% of CMML patients at diagnosis. Spectrum of gene mutations does not differ from the ones reported in more heterogeneous CMML cohorts [6, 7, 11, 15]. This study confirms the molecular heterogeneity of the disease. In addition, in the current study, we report for the first time recurrent mutations (5-10%) in CMML in the genes *CREBBP*, *KMT2D* and *UMODL1*, which have been previously reported in lymphoid neoplasms or solid tumors [25, 26]. Studies in larger cohorts should provide more insights in the involvement of these genes in the pathogenesis of CMML. On the other hand, although multiple mutations in different genes can be detected in most of CMML patients, it is interesting to point out that 91% of patients are characterized by harboring a mutation in at least one of the three most recurrent genes in CMML (*TET2*, *ASXL1*, *SRSF2*), which can be used for diagnostic purposes.

Across the entire cohort, 14/48 (29.2%) patients presented with interstitial CNN-LOH, as previously reported by our group [18]. All patients with CNN-LOH in 4q24 harbored a *TET2* mutation and all cases with CNN-LOH in 11q23.3 presented with a *CBL* mutation, confirming the association between both types of molecular events [27, 28]. Similarly, single patients with *EZH2*, *NRAS* and *SRSF2* mutations also presented CNN-LOH in 7q35-q36, 12p12.1 and 17q25.3, respectively, suggesting that detection of CNN-LOH in CMML indicates the presence of homozygous mutations in genes located in the affected region. The negative impact of CNN-LOH observed in CMML [18] may be influenced or even enhanced by the presence of mutations in genes located in these regions.

Targeted sequencing was also performed in seven patients at time of AML progression and revealed the acquisition of additional mutations in 6/7 (85.7%) patients, all of them with at least one mutation in a gene involved in cell signaling. Five of these patients acquired mutations in components or regulators of the RAS signaling pathway (*KRAS, NRAS, PTPN1, BRAF* and *FLT3*), suggesting that activation of RAS pathway is probably involved in the evolution of CMML in some patients. Mutations in these genes have been previously associated with CMML-MP [14, 23]. The remaining patient acquired a mutation in *GNAS*, a gene involved in the GPCR signaling pathway. Somatic activating mutations in *GNAS* are common in solid tumors but were recently identified for the first time in hematological neoplasms, in 1% of MDS [29]. Implication of *GNAS* mutations in MDS or related myeloid neoplasms has not been further investigated. In our study, mutations in *ASXL1* and *TET2*wt at diagnosis correlated with AML progression. This association has already been reported for *ASXL1* mutations [14, 15]. Thus, even though acquisition of cell signaling mutations involved in AML progression cannot be anticipated, presence of other mutations at diagnosis may predict AML transformation and serve as prognostic markers for a closer monitoring of these patients or to be considered as candidates to a more aggressive treatment.

Correlation analyses between clinical and biological features and gene mutations revealed interesting associations. Of note, adverse risk genes such as *ASXL1*, *EZH2* and *TET2*wt, were in many cases associated with high risk parameters, such as CMML-2, lower Hg levels, higher leukocyte counts, higher risk groups of CPSS and GFM models or progression to AML. In addition, as expected, myeloproliferative features such as CMML-MP variant, leukocyte count or presence of splenomegaly, correlated with genes involved in cell signaling, such as *NRAS* and *JAK2*.

The prognostic value of individual gene mutations was explored by performing survival analyses and investigating correlation with clinical features. Mutations in *ASXL1*, *EZH2*, *NRAS* and *SRSF2* correlated with different clinical or biological features that are known to be associated with worse outcome in CMML [15–17]. Mutations in these four genes were associated with shorter OS in univariate survival analyses and all, except from *SRSF2*, correlated with shorter PFS as well. Some of these associations have already been reported, but *ASXL1* is the only marker that has been shown to be prognostically detrimental on multivariate models [6, 15, 24]. Therefore, impact on presenting one or more adverse risk gene mutations (*ASXL1, EZH2, NRAS, SRSF2*) was investigated. Presence of mutations in at least one of these genes was predictive of both OS and PFS and was the only variant that remained significant in OS multivariate model in addition to CPSS. Of note, number of adverse risk gene mutations was also predictive of OS and PFS when patients were classified in three (0, 1, ≥2) and four (0, 1, 2, ≥3) subgroups, which suggests an additive negative impact of presenting mutations in these genes. Interestingly, survival outcomes were also affected by the number of total concurrent mutations, meaning that each acquired mutation confers an additional detrimental value, as has been reported in MDS and MPN [12, 30]. Focusing on PFS, *TET2*wt was also predictive of shorter PFS, supporting the favorable impact of *TET2* mutations that has been previously reported but remains controversial [5, 13, 27, 28]. Of note, we detected *TET2* mutations at a higher frequency than the one reported in other studies [31-33]. Considering that *TET2* mutations are associated with good prognosis features and may play a protective role in CMML, this higher frequency could be explained due to the fact that our cohort of patients is focused on lower risk CMML. Patnaik *et al* recently reported a prognostic interaction between *ASXL1* and *TET2* mutations, and suggested a favorable impact from *TET2* mutations in the absence of *ASXL1* mutations [6]. We investigated this interaction in our cohort and observed that the classification of patients according to the status of these two genes was associated with OS and PFS. Even though the four survival curves did not match the ones reported by Patnaik *et al*, probably due to the limited number of patients in our cohort in the middle subgroups (*TET2*wt-*ASXL1*mut and *TET2*wt-*ASXL1*wt), we did confirm the significant negative impact of *ASXL1* mutations and the favorable impact of *TET2* mutations in the absence of *ASXL1* mutations. Interestingly, the group of *TET2*wt-*ASXL1*wt had a quite unfavorable prognosis, worse than the *TET2*-wt/*ASXL1*-mut, which is what Patnaik *et al* described and what would be expected. This could be explained because some of the patients in the *TET2*wt-*ASXL1*wt group (n=7) carry mutations in other adverse risk genes. Specifically, two of them carried *SRSF2* mutations and two carried *RUNX1* mutations, which also have been reported to have an adverse prognostic impact, even though this was not confirmed in our cohort [15]. The prognostic interaction between *TET2* and other adverse risk gene mutations was also analyzed. The analysis confirmed that the negative impact of mutations in adverse risk genes prevails over *TET2* mutations, although patients with *TET2* mutations have a better outcome compared to patients without, suggesting a protective role for *TET2* mutations. The analysis also revealed that in the absence of adverse risk gene mutations, *TET2* mutations confer the best outcome. This beneficial impact of *TET2* mutations was even more noticeable in the PFS analysis, which Patnaik *et al* did not report. Furthermore, *TET2*wt was the only significant variant in the PFS multivariate model in addition to CPSS.

Although our series is limited by the number of samples compared to other series, it is the only one mainly focused on CMML patients with low risk cytogenetic features. In addition, the results of multivariate analyses for both OS and PFS suggest that our findings may be applicable to larger series of patients. Our multivariate model confirms the prognostic impact of the CPSS scoring system for both OS and PFS and implies that molecular studies can add prognostic value to this model, especially in patients with low risk cytogenetic features (normal karyotype, isolated -Y) or uninformative CC.

In summary, we report mutations in nearly all patients with CMML and low risk cytogenetic features, some of which have a negative impact on the outcome of patients. With NGS technologies being more accessible each day, we would recommend to perform targeted molecular analysis of *ASXL1* and, if possible, *EZH2*, *NRAS*, *SRSF2* and *TET2* in patients with CMML and low risk cytogenetic features or uninformative CC. This may allow to identify patients that are more likely to present with an aggressive disease evolution and that could benefit from closely monitoring and more intensive treatments.

## MATERIALS AND METHODS

### Patients and samples

A retrospective study was performed on a total of 56 patients with CMML from *Institut Català d’Oncologia* (ICO). Patients were diagnosed according to the FAB [2] and 2008 WHO [1] classifications. Cases with CMML and low risk cytogenetic features (normal karyotype or isolated -Y) or no metaphases at diagnosis were included in the study. Study approval was obtained from *ICO-Hospital Germans Trias i Pujol* Ethics Committee. Informed consent was obtained from all patients, in accordance with the Declaration of Helsinki.

### Cytogenetics

Conventional G-banding cytogenetics was performed on bone marrow samples at diagnosis following standard procedures [5]. We analyzed 20 metaphases per sample (n=54) except in two cases in which no metaphases were obtained. Karyotypes were described according to the International System for Human Cytogenetic Nomenclature 2013 [34].

### DNA samples

Samples were collected at diagnosis for all patients and at time of AML progression in 7 patients and CMML-2 progression in one case. Whole bone marrow samples (n= 58) or peripheral blood (n=6) were used. Genomic DNA was extracted with QiaAmp DNA Blood Mini kit (Qiagen, Hilden, Germany) and quantified using Quant-iT PicoGreen dsDNA Assay Kit (Invitrogen, CA, USA).

### Targeted deep sequencing

Targeted deep-sequencing of a panel of 83 myeloid-related genes was performed in all samples (Supplementary Table S4). Indexed libraries were prepared with 1μg of double strand genomic DNA using the Kapa Library Preparation Kit (Kapa Biosystems, MA, USA). Custom target capture enrichment using the SeqCap EZ capture chemistry (Nimblegen, Roche, Basel, Switzerland) was performed on pools of 8 libraries. Multiplexed captured libraries were sequenced on an Illumina MiSeq following a 150bp paired-end reads standard protocol.

### Targeted sequencing data analysis

Sequencing data were analyzed using an in-house pipeline. Reads were aligned against human genome build 19 (hg19) using BWA 0.7.12 [35]. Post-alignment including local indel realignment and base recalibration was performed using the tools in GATK 3.4.46 software package [36]. Packages SAMtools 1.2 and VarScan 2.4.0 were used for variant calling and ANNOVAR (version 2015Jun17) for variant annotation [37, 38]. High-probability oncogenic mutations were called by eliminating sequencing and mapping errors and by discarding variants located in highly variable regions or with low coverage, as well as SNPs described on the available databases and synonymous variants. Variants were also filtered according to the variant allele frequency (VAF): all variants with VAF ≥5% were reported, as well as variants with VAF<5% and at least 25 reads for the variant that are known hotspots and have been reported in hematological neoplasms.

### Statistical analysis

Baseline characteristics were described as frequency and percentage for categorical variables and median and range for quantitative variables. Comparisons of categorical variables between patient subsets were compared using χ^2^ or Fisher's exact test, when appropriate, while median test was used to compare continuous variables. Comparisons of paired data for continuous variables were performed with the Wilcoxon test. OS was defined as time from diagnosis to the last follow-up or death from any cause and PFS as time from diagnosis to progression to AML (presence of ≥20% of blasts in bone marrow or peripheral blood) or death from CMML [39]. Survival curves were calculated using the Kaplan-Meier method and log-rank test was used for comparisons between groups. Multivariate analysis was performed using Cox proportional-hazards regression model, considering Wald Backward as selection method. Two-sided *P* values <0.05 were considered as statistically significant. The statistical package SPSS, version 23.0 (SPSS Inc., Chicago, IL, USA) was used for all analyses.

## SUPPLEMENTARY MATERIALS FIGURES AND TABLES




