# Supplementary material for: Targeted deep sequencing improves outcome stratification in chronic myelomonocytic leukemia with low risk cytogenetic features
Source: Oncotarget. 2016 Jul 29;7(35):57021–35. doi: 10.18632/oncotarget.10937 (PMC5302970; doi:10.18632/oncotarget.10937)
Supplement: Supplementary file 1 [file oncotarget-07-57021-s001.pdf]

Targeted deep sequencing improves outcome stratification in chronic myelomonocytic leukemia with low risk cytogenetic features

SUPPLEMENTARY FIGURES AND TABLES

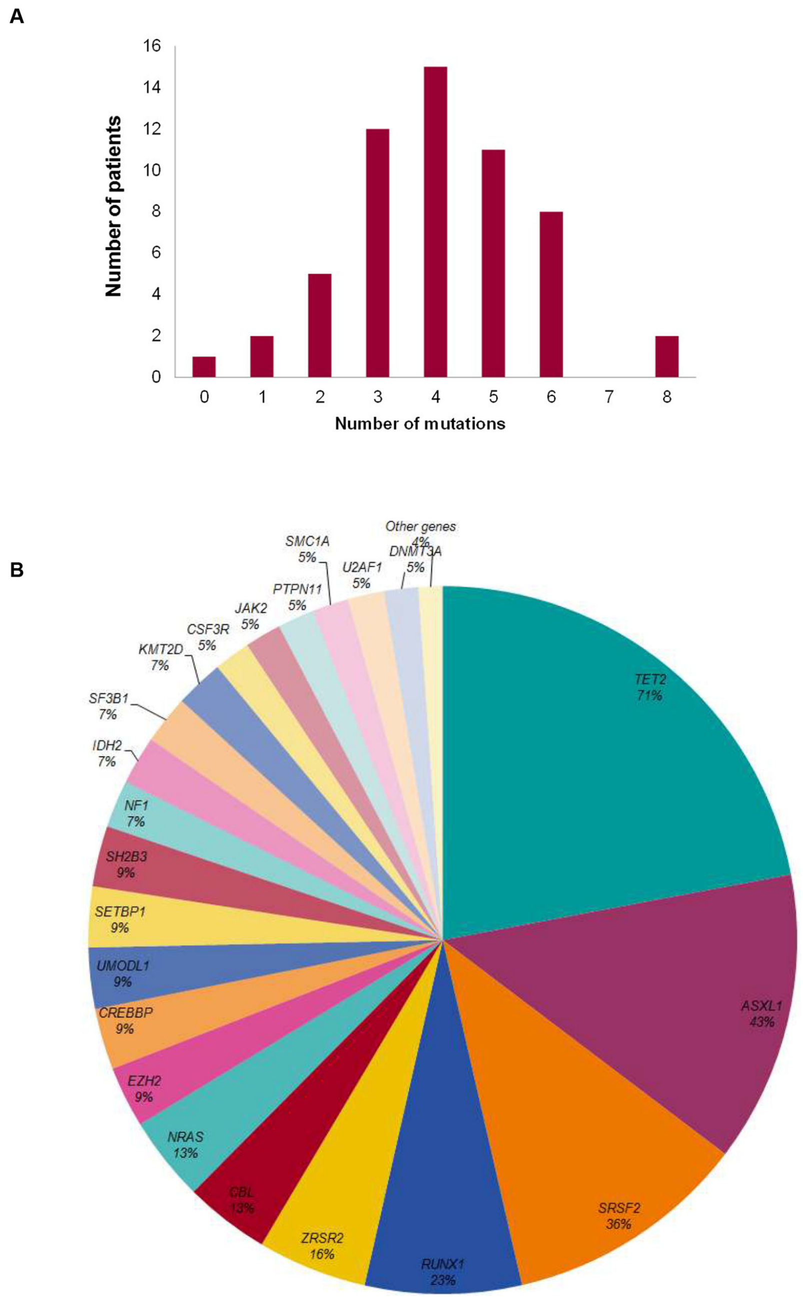

Supplementary Figure S1: Number and type of mutations across the CMML patients at diagnosis. A. distribution of number of mutations detected per patient; **B.** frequency of affected genes in the entire cohort. (*Continued*)

C

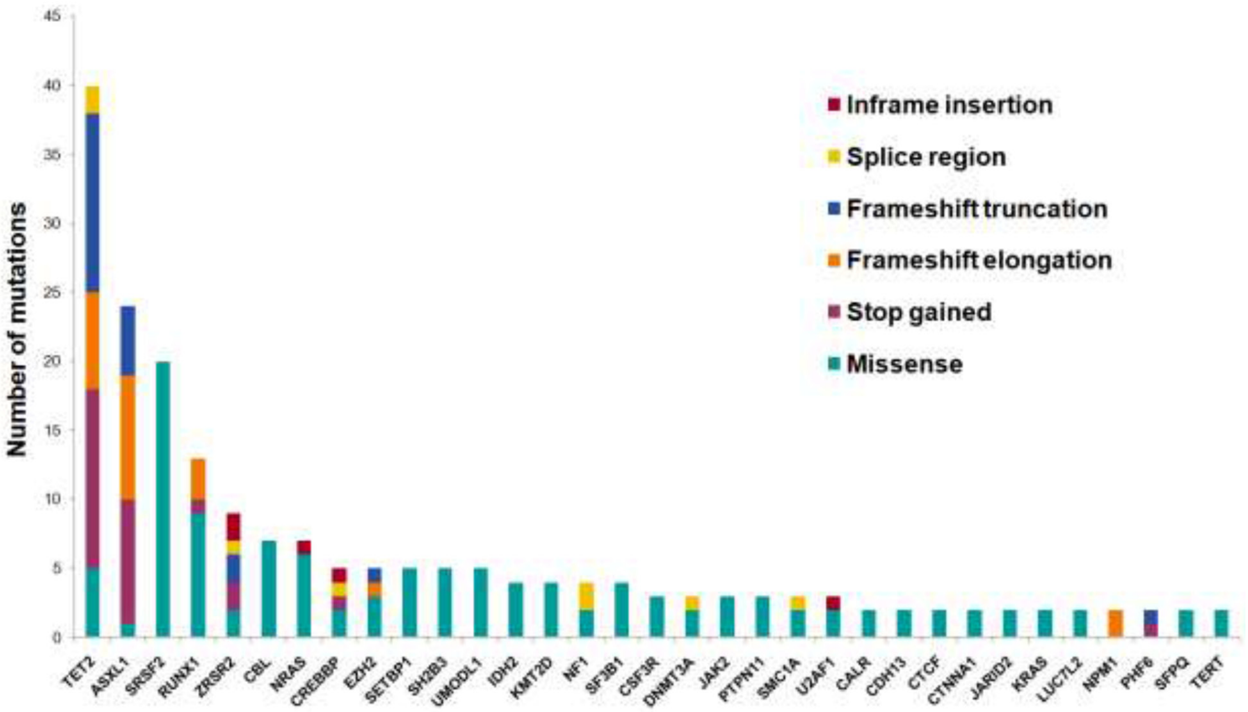

D

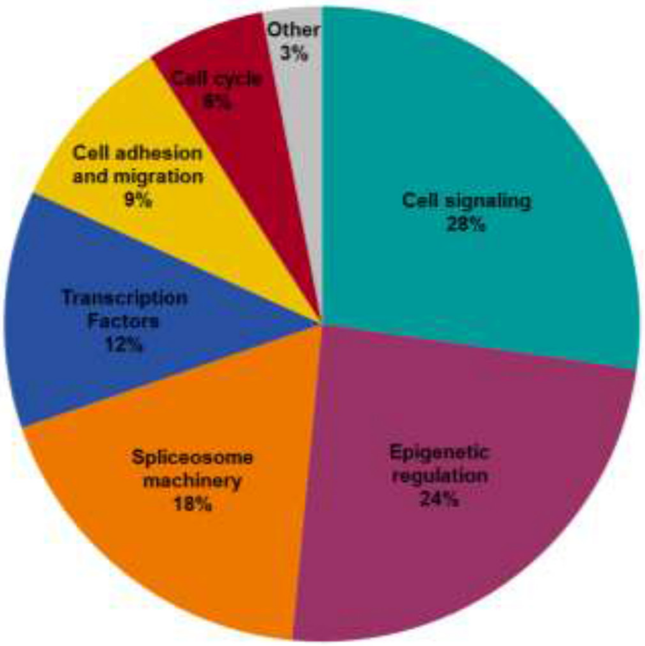

Supplementary Figure S1: (*Continued*) Number and type of mutations across the CMML patients at diagnosis. C. type of mutation; **D.** mechanisms in which the main affected genes are involved.

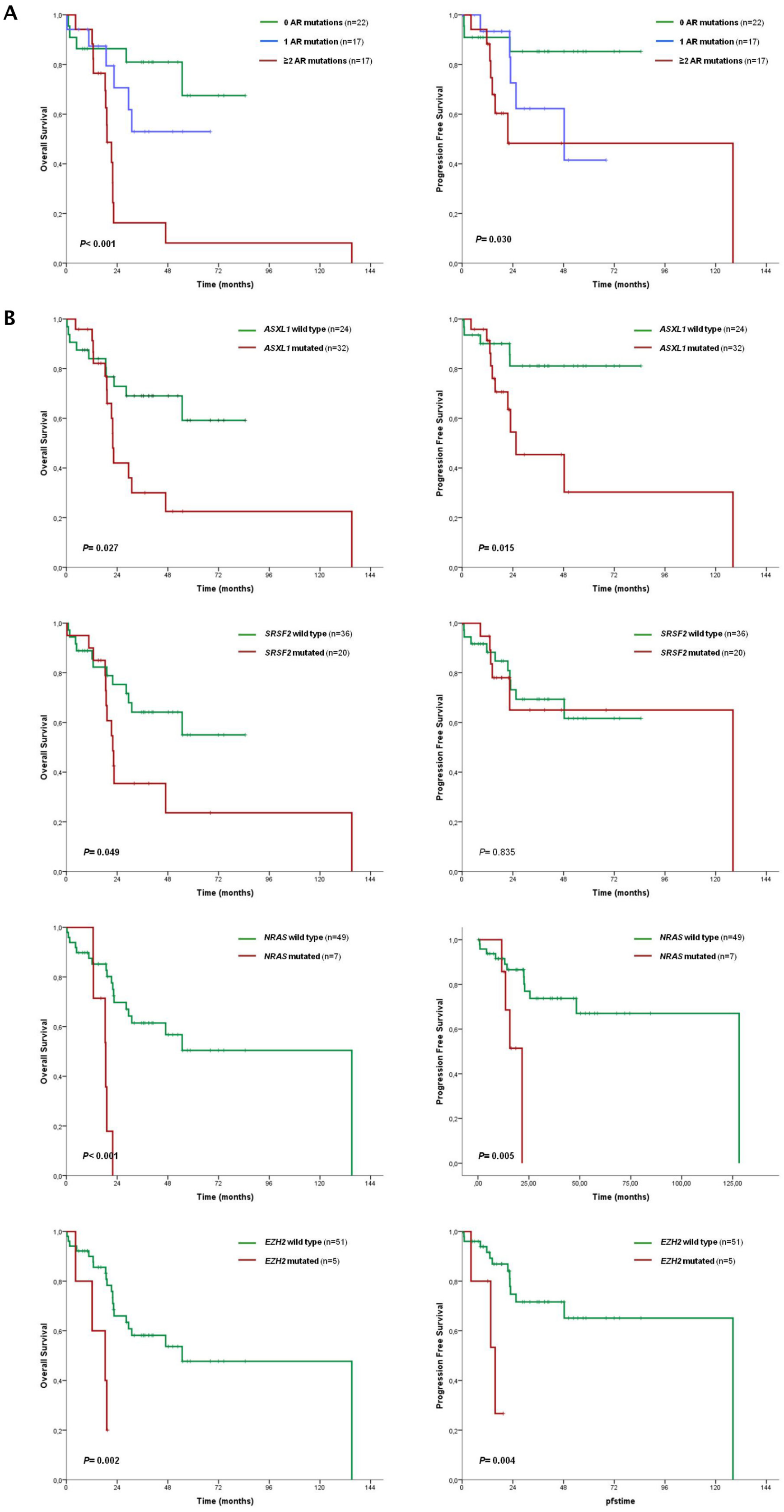

Supplementary Figure S2: Prognostic impact of gene mutations. A. OS and PFS curves according to number of total mutations; **B.** OS and PFS curves according to mutations in individual genes (*ASXL1*, *EZH2, NRAS, SRSF2* and *TET2*). See Table 3 for 3-year percentage overall survival and progression free survival and confidence intervals. (*Continued*)

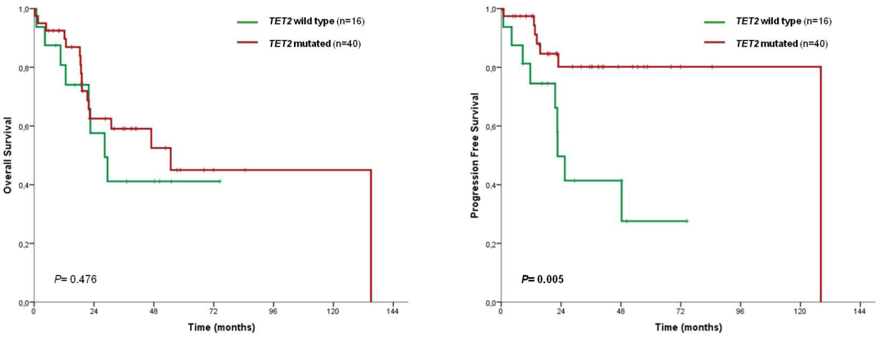

C

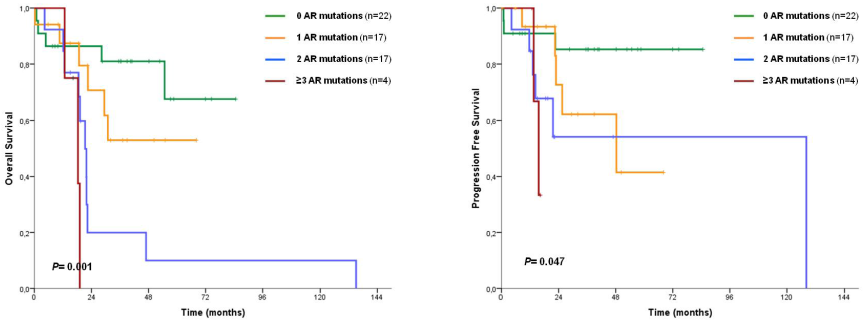

D

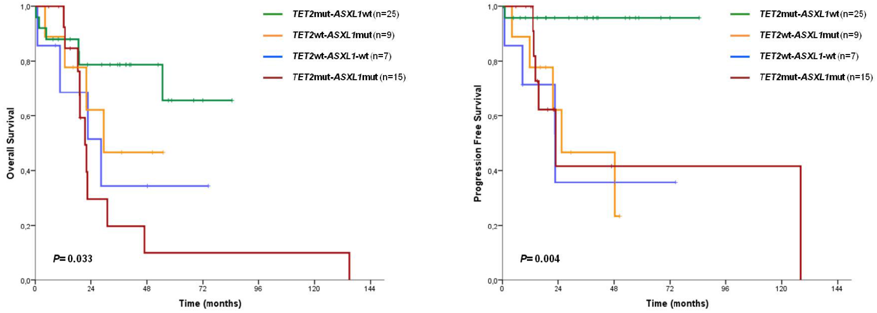

E

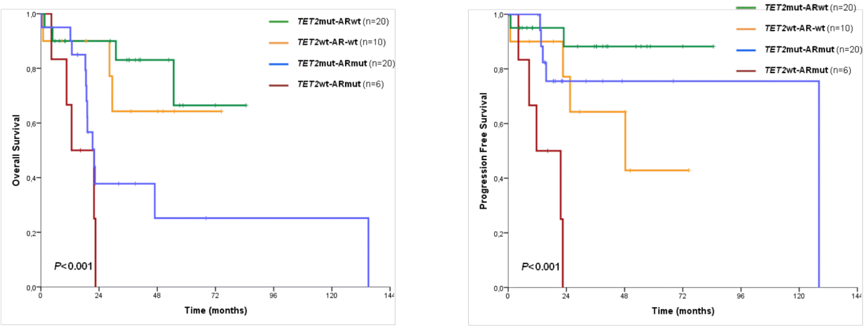

Supplementary Figure S2: (*Continued*) Prognostic impact of gene mutations. C. OS and PFS curves according to number of adverse risk gene mutations (*ASXL1*, *EZH2, NRAS*, *SRSF2*); **D.** OS and PFS curves according to combinations between *ASXL1* and *TET2* mutations. AR mutations: adverse risk gene mutations (*ASXL1*, *EZH2*, *NRAS, SRSF2*). **E.** OS and PFS curves according to combinations between *TET2* mutations and adverse risk genes (excluding *ASXL1*). AR mutations excluding *ASXL1* (*EZH2*, *NRAS, SRSF2*). See Table 3 for 3-year percentage overall survival and progression free survival and confidence intervals.

Supplementary Table S1: Detected variants in the whole cohort of CMML patients at diagnosis (n=56).

See Supplementary File 1

Supplementary Table S2: List of all the affected genes and frequency in the cohort of CMML patients in samples at diagnosis (n=56)

Gene	Number of patients	Cohort frequency	Gene	Number of patients	Cohort frequency
TET2	40	71%	*PHF6*	2	4%
ASXL1	24	43%	*SFPQ*	2	4%
SRSF2	20	36%	*TERT*	2	4%
RUNX1	13	23%	*MECOM*	1	2%
ZRSR2	9	16%	*ATRX*	1	2%
CBL	7	13%	*BCOR*	1	2%
NRAS	7	13%	*CDH3*	1	2%
EZH2	5	9%	*BRAF*	1	2%
CREBBP	5	9%	*CUX1*	1	2%
UMODL1	5	9%	*EP300*	1	2%
SETBP1	5	9%	*FLT3*	1	2%
SH2B3	5	9%	*GATA1*	1	2%
NF1	4	7%	*GCAT*	1	2%
IDH2	4	7%	*GATA2*	1	2%
SF3B1	4	7%	*IDH1*	1	2%
KMT2D	4	7%	*KIT*	1	2%
CSF3R	3	5%	*KMT2A*	1	2%
JAK2	3	5%	*PDGFRA*	1	2%
PTPN11	3	5%	*PDGFRB*	1	2%
SMC1A	3	5%	*PHLPP1*	1	2%
U2AF1	3	5%	*RAD21*	1	2%
DNMT3A	3	5%	*RPS14*	1	2%
KRAS	2	4%	*SF1*	1	2%
CTNNA1	2	4%	*SF3A1*	1	2%
CDH13	2	4%	*SMC3*	1	2%
CTCF	2	4%	*STAG2*	1	2%
CALR	2	4%	*SUZ12*	1	2%
JARID2	2	4%	*TIMM50*	1	2%
LUC7L2	2	4%	*AEBP2*	1	2%
NPM1	2	4%			

Supplementary Table S3: Patients with gene mutations in regions with copy number neutral loss of heterozygosity (CNN-LOH)

Number of patients	CNN-LOH region	Start	End	Size	Mutated gene
4	4q13.3q35.2	70579280	190921709	*120342429*	*TET2*
	4q13.3q35.2	89992346	190921709	100.929.363	
	4q13.3qter	111.229.207	79.692.502	190.921.709	
	4q13.3qter	108.964.973	81.956.736	190.921.709	
3	11q13.3q25	65018466	134942626	69.924.160	*CBL*
	11q13.2q25	67.393.850	134938470	67.544.620	
	11q13.2q25	*70339930*	134939692	*64599762*	
1	7q22.1q36.3	98915957	159119220	60.203.263	*EZH2*
1	17q25.3	78.966.914	81.041.938	2.075.024	*SRSF2*
1	12q21.2q24.33	75.863.034	133777902	57.914.868	*KRAS*
1	7p12.3q21.11	45749533	77814597	32.065.064	None of the studied genes
1	10p12.1q21.1	29074038	57196819	28.122.781	
1	13q14.11q31.3	43719771	93333822	49.614.051	
1	14q11.2q21.3	20511672	50870199	30.358.527	

Supplementary Table S4: Genes included in the 83 gene panel

Gene	Target region (exon)	Gene	Target region (exon)	Gene	Target region (exon)	Gene	Target region (exon)
ABL1	4-9	*EED*	full	*MECOM*	full	*SF3B1*	10-16
AEBP2	full	*EP300*	full	*KMT2A*	full	*SFPQ*	full
ASXL1	9, 11, 12	*ETV6*	full	*MLL2*	full	*SH2B3*	full
ATRX	full	*EZH2*	full	*MPL*	10	*SMC1A*	full
BCOR	full	*FLT3*	14, 15, 20	*NF1*	full	*SMC3*	full
BCORL1	full	*GATA1*	2	*NPM1*	11, 12	*SPARC*	full
BRAF	full	*GATA2*	full	*NRAS*	1-3	*SRSF2*	1
CALR	9	*GCAT*	full	*PDGFRA*	full	*STAG1*	full
CBL	8, 9	*GNAS*	full	*PDGFRB*	full	*STAG2*	full
CBLB	9, 10	*HRAS*	2, 3	*PHF6*	full	*SUZ12*	full
CDH13	full	*IDH1*	4	*PHLPP1*	full	*TERC*	full
CDH3	full	*IDH2*	4	*PTEN*	5-8	*TERT*	full
CDKN2A	full	*IKZF1*	full	*PTPN11*	full	*TET2*	2-11
CEBPA	full	*IRF1*	full	*RAD21*	full	*TGM2*	full
CREBBP	full	*JAK2*	12-16	*RPS14*	full	*TIMM50*	full
CSF3R	full	*JAK3*	13	*RUNX1*	3-8	*TP53*	4-11
CSNK1A1	full	*JARID2*	full	*SALL4*	full	*U2AF1*	2, 6
CTCF	full	*KDM6A*	full	*SBDS*	full	*UMODL1*	full
CTNNA1	full	*KIT*	2,8-11,13,17	*SETBP1*	4	*WT1*	7, 9
CUX1	full	*KRAS*	1-3	*SF1*	full	*ZRSR2*	full
DNMT3A	full	*LUC7L2*	full	*SF3A1*	full		
