# Supplementary material for: Targeted deep sequencing improves outcome stratification in chronic myelomonocytic leukemia with low risk cytogenetic features
Source: Oncotarget. 2016 Jul 29;7(35):57021–35. doi: 10.18632/oncotarget.10937 (PMC5302970; doi:10.18632/oncotarget.10937)

**SUPPLEMENTARY DATA**

**Supplementary Tables**

**Supplementary Table 1**. Detected variants in the whole cohort of CMML patients at diagnosis (n=56)

| **CMML** | **Gene** | **Variant** | **Chr** | **Coordinate** | **Type** | **VAF(%)** |
| --- | --- | --- | --- | --- | --- | --- |
| 62 | *AEBP2* | G>G/C | 12 | 19592744 | snv | 14 |
| 4 | *ASXL1* | A>A/G | 20 | 31024260 | snv | 49 |
| 4 | *ASXL1* | TCACCACTGCCATAGAGAGGCGGC>TCACCACTGCCATAGAGAGGCGGC/T | 20 | 31022402 | deletion | 6 |
| 4 | *ASXL1* | GA>GA/G | 20 | 31022416 | deletion | 9 |
| 6 | *ASXL1* | TC>TC/T | 20 | 31022898 | deletion | 45 |
| 7 | *ASXL1* | T>T/A | 20 | 31022615 | snv | 28 |
| 7 | *ASXL1* | G>G/T | 20 | 31023821 | snv | 56 |
| 9 | *ASXL1* | T>T/TA | 20 | 31022613 | insertion | 46 |
| 10 | *ASXL1* | C>C/T | 20 | 31022592 | snv | 52 |
| 13 | *ASXL1* | G>G/T | 20 | 31024662 | snv | 37 |
| 16 | *ASXL1* | A>A/AG | 20 | 31022441 | insertion | 35 |
| 17 | *ASXL1* | A>A/AG | 20 | 31022441 | insertion | 43 |
| 19 | *ASXL1* | C>C/T | 20 | 31022589 | snv | 43 |
| 27 | *ASXL1* | A>A/AG | 20 | 31022441 | insertion | 37 |
| 32 | *ASXL1* | C>C/T | 20 | 31022851 | snv | 46 |
| 34 | *ASXL1* | A>A/AG | 20 | 31022441 | insertion | 42 |
| 41 | *ASXL1* | C>C/T | 20 | 31024758 | snv | 49 |
| 48 | *ASXL1* | A>A/AG | 20 | 31022441 | insertion | 38 |
| 50 | *ASXL1* | A>A/AG | 20 | 31022441 | insertion | 43 |
| 53 | *ASXL1* | CTG>CTG/C | 20 | 31021542 | deletion | 43 |
| 60 | *ASXL1* | A>A/AG | 20 | 31022441 | insertion | 34 |
| 61 | *ASXL1* | G>G/T | 20 | 31022697 | snv | 47 |
| 64 | *ASXL1* | GA>GA/G | 20 | 31022416 | deletion | 12 |
| 64 | *ASXL1* | TCACCACTGCCATAGAGAGGCGGC>TCACCACTGCCATAGAGAGGCGGC/T | 20 | 31022402 | deletion | 6 |
| 86 | *ASXL1* | GA>GA/G | 20 | 31022416 | deletion | 12 |
| 86 | *ASXL1* | TCACCACTGCCATAGAGAGGCGGC>TCACCACTGCCATAGAGAGGCGGC/T | 20 | 31022402 | deletion | 6 |
| 114 | *ASXL1* | C>C/T | 20 | 31022592 | snv | 50 |
| 115 | *ASXL1* | C>C/T | 20 | 31022637 | snv | 35 |
| 116 | *ASXL1* | G>G/T | 20 | 31022487 | snv | 44 |
| 117 | *ASXL1* | A>A/AG | 20 | 31022441 | insertion | 24 |
| 37 | *ATRX* | A>A/G | X | 76938584 | snv | 100 |
| 31 | *BCOR* | G>G/A | X | 39932591 | snv | 100 |
| 27 | *BRAF* | T>T/C | 7 | 140534599 | snv | 49 |
| 18 | *CALR* | A>A/G | 19 | 13054612 | snv | 51 |
| 62 | *CALR* | A>A/G | 19 | 13054612 | snv | 51 |
| 6 | *CBL* | G>G/A | 11 | 119148922 | snv | 32 |
| 17 | *CBL* | T>T/C | 11 | 119148919 | snv | 24 |
| 28 | *CBL* | G>G/A | 11 | 119148991 | snv | 90 |
| 34 | *CBL* | G>G/T | 11 | 119149003 | snv | 2 |
| 46 | *CBL* | G>G/A | 11 | 119148991 | snv | 2 |
| 53 | *CBL* | G>G/C | 11 | 119148967 | snv | 36 |
| 62 | *CBL* | G>G/A | 11 | 119149251 | snv | 3 |
| 61 | *CDH13* | C>C/G | 16 | 83520115 | snv | 48 |
| 62 | *CDH13* | G>G/A | 16 | 83065791 | snv | 55 |
| 4 | *CDH3* | C>C/T | 16 | 68732113 | snv | 40 |
| 7 | *CREBBP* | G>G/A | 16 | 3779482 | snv | 26 |
| 26 | *CREBBP* | T>T/C | 16 | 3777826 | snv | 47 |
| 36 | *CREBBP* | G>G/A | 16 | 3820773 | snv | 46 |
| 38 | *CREBBP* | C>C/CGCTGCT | 16 | 3778302 | insertion | 18 |
| 62 | *CREBBP* | C>C/G | 16 | 3781195 | snv | 47 |
| 10 | *CSF3R* | C>C/T | 1 | 36932047 | snv | 51 |
| 27 | *CSF3R* | C>C/T | 1 | 36932047 | snv | 49 |
| 116 | *CSF3R* | C>C/G | 1 | 36939403 | snv | 50 |
| 29 | *CTCF* | G>G/A | 16 | 67654676 | snv | 28 |
| 46 | *CTCF* | G>G/A | 16 | 67650711 | snv | 23 |
| 33 | *CTNNA1* | G>G/C | 5 | 138266577 | snv | 48 |
| 123 | *CTNNA1* | G>G/C | 5 | 138269550 | snv | 52 |
| 116 | *CUX1* | G>G/A | 7 | 101891907 | snv | 52 |
| 15 | *DNMT3A* | C>C/T | 2 | 25457242 | snv | 46 |
| 18 | *DNMT3A* | C>C/T | 2 | 25466759 | snv | 51 |
| 34 | *DNMT3A* | T>T/G | 2 | 25523096 | snv | 46 |
| 17 | *EP300* | TCAG>TCAG/T | 22 | 41574510 | deletion | 44 |
| 10 | *EZH2* | G>G/GCTTC | 7 | 148544339 | insertion | 39 |
| 17 | *EZH2* | GT>GT/G | 7 | 148515160 | deletion | 45 |
| 50 | *EZH2* | C>G/G | 7 | 148525940 | snv | 99 |
| 86 | *EZH2* | T>T/A | 7 | 148507437 | snv | 34 |
| 114 | *EZH2* | T>T/C | 7 | 148506166 | snv | 12 |
| 25 | *FLT3* | T>T/C | 13 | 28608312 | snv | 47 |
| 48 | *GATA1* | C>C/T | X | 48649629 | snv | 99 |
| 3 | *GATA2* | T>T/TG | 3 | 128205798 | insertion | 46 |
| 30 | *GCAT* | C>C/T | 22 | 38211772 | snv | 46 |
| 38 | *IDH1* | G>G/A | 2 | 209113113 | snv | 48 |
| 28 | *IDH2* | C>C/T | 15 | 90631934 | snv | 49 |
| 32 | *IDH2* | C>C/T | 15 | 90631934 | snv | 45 |
| 64 | *IDH2* | C>C/T | 15 | 90631934 | snv | 47 |
| 111 | *IDH2* | C>C/T | 15 | 90631934 | snv | 2 |
| 25 | *JAK2* | G>G/T | 9 | 5073770 | snv | 48 |
| 26 | *JAK2* | G>G/T | 9 | 5073770 | snv | 44 |
| 50 | *JAK2* | G>G/T | 9 | 5073770 | snv | 28 |
| 8 | *JARID2* | G>G/A | 6 | 15497053 | snv | 51 |
| 33 | *JARID2* | A>A/G | 6 | 15496802 | snv | 58 |
| 41 | *KIT* | A>A/T | 4 | 55599321 | snv | 48 |
| 83 | *KMT2A* | G>G/A | 11 | 118374244 | snv | 52 |
| 16 | *KMT2D* | G>G/T | 12 | 49426793 | snv | 48 |
| 38 | *KMT2D* | C>C/T | 12 | 49433050 | snv | 53 |
| 63 | *KMT2D* | G>G/A | 12 | 49433958 | snv | 53 |
| 84 | *KMT2D* | C>C/A | 12 | 49426734 | snv | 46 |
| 1 | *KRAS* | C>C/A | 12 | 25398262 | snv | 10 |
| 62 | *KRAS* | C>C/T | 12 | 25398285 | snv | 42 |
| 46 | *LUC7L2* | A>A/C | 7 | 139107009 | snv | 47 |
| 86 | *LUC7L2* | A>A/C | 7 | 139107009 | snv | 49 |
| 25 | *MECOM* | C>C/A | 3 | 168861536 | snv | 50 |
| 1 | *NF1* | T>T/C | 17 | 29557853 | snv | 8 |
| 60 | *NF1* | T>T/C | 17 | 29557853 | snv | 6 |
| 63 | *NF1* | G>G/A | 17 | 29562747 | snv | 4 |
| 116 | *NF1* | G>G/A | 17 | 29548916 | snv | 44 |
| 116 | *NF1* | G>G/T | 17 | 29586074 | snv | 41 |
| 8 | *NPM1* | C>C/CTCTG | 5 | 170837543 | insertion | 31 |
| 15 | *NPM1* | C>C/CTCTG | 5 | 170837543 | insertion | 30 |
| 1 | *NRAS* | C>C/T | 1 | 115258747 | snv | 32 |
| 4 | *NRAS* | A>A/C | 1 | 115256521 | snv | 15 |
| 4 | *NRAS* | C>C/T | 1 | 115258744 | snv | 6 |
| 6 | *NRAS* | T>T/TTAG | 1 | 115258676 | insertion | 11 |
| 9 | *NRAS* | C>C/T | 1 | 115258747 | snv | 30 |
| 10 | *NRAS* | C>C/A | 1 | 115256532 | snv | 47 |
| 50 | *NRAS* | C>C/T | 1 | 115258747 | snv | 5 |
| 64 | *NRAS* | C>C/T | 1 | 115258747 | snv | 53 |
| 15 | *PDGFRA* | A>A/T | 4 | 55127309 | snv | 50 |
| 36 | *PDGFRB* | C>C/T | 5 | 149512494 | snv | 47 |
| 84 | *PHF6* | C>C/T | X | 133551319 | snv | 46 |
| 121 | *PHF6* | TG>TG/T | X | 133511705 | deletion | 73 |
| 121 | *PHF6* | G>G/T | X | 133511706 | snv | 20 |
| 122 | *PHLPP1* | C>C/T | 18 | 60646006 | snv | 49 |
| 4 | *PTPN11* | G>G/A | 12 | 112926888 | snv | 6 |
| 28 | *PTPN11* | G>G/A | 12 | 112926887 | snv | 3 |
| 63 | *PTPN11* | G>G/A | 12 | 112926888 | snv | 23 |
| 15 | *RAD21* | C>C/T | 8 | 117878966 | snv | 53 |
| 35 | *RPS14* | T>T/C | 5 | 149823902 | snv | 47 |
| 6 | *RUNX1* | C>C/A | 21 | 36231773 | snv | 33 |
| 6 | *RUNX1* | G>G/T | 21 | 36252940 | snv | 12 |
| 27 | *RUNX1* | T>T/TCC | 21 | 36252994 | insertion | 3 |
| 28 | *RUNX1* | A>A/G | 21 | 36259324 | snv | 45 |
| 34 | *RUNX1* | C>C/CG | 21 | 36259256 | insertion | 38 |
| 38 | *RUNX1* | C>C/T | 21 | 36259171 | snv | 43 |
| 39 | *RUNX1* | A>A/G | 21 | 36259324 | snv | 52 |
| 41 | *RUNX1* | G>G/A | 21 | 36206725 | snv | 42 |
| 45 | *RUNX1* | G>G/A | 21 | 36259184 | snv | 46 |
| 50 | *RUNX1* | AG>AG/A | 21 | 36206765 | deletion | 46 |
| 50 | *RUNX1* | T>T/C | 21 | 36252886 | snv | 51 |
| 60 | *RUNX1* | C>C/T | 21 | 36231792 | snv | 45 |
| 62 | *RUNX1* | A>A/G | 21 | 36259324 | snv | 50 |
| 63 | *RUNX1* | A>A/G | 21 | 36259324 | snv | 52 |
| 83 | *RUNX1* | G>G/A | 21 | 36231786 | snv | 9 |
| 3 | *SETBP1* | C>C/T | 18 | 42531184 | snv | 45 |
| 9 | *SETBP1* | A>A/G | 18 | 42531908 | snv | 44 |
| 34 | *SETBP1* | G>G/A | 18 | 42533267 | snv | 50 |
| 45 | *SETBP1* | G>G/C | 18 | 42530117 | snv | 47 |
| 82 | *SETBP1* | C>C/T | 18 | 42531184 | snv | 54 |
| 37 | *SF1* | AGGC>AGGC/A | 11 | 64534502 | deletion | 41 |
| 29 | *SF3A1* | C>C/A | 22 | 30733688 | snv | 43 |
| 3 | *SF3B1* | T>T/C | 2 | 198266834 | snv | 48 |
| 21 | *SF3B1* | T>T/C | 2 | 198266834 | snv | 47 |
| 82 | *SF3B1* | T>T/C | 2 | 198266834 | snv | 4 |
| 117 | *SF3B1* | T>T/G | 2 | 198267360 | snv | 3 |
| 30 | *SFPQ* | G>G/A | 1 | 35658085 | snv | 53 |
| 114 | *SFPQ* | A>A/G | 1 | 35658319 | snv | 48 |
| 3 | *SH2B3* | G>G/A | 12 | 111856640 | snv | 43 |
| 46 | *SH2B3* | G>G/A | 12 | 111885287 | snv | 34 |
| 84 | *SH2B3* | G>G/A | 12 | 111856607 | snv | 43 |
| 86 | *SH2B3* | G>G/C | 12 | 111856571 | snv | 51 |
| 122 | *SH2B3* | C>C/T | 12 | 111885484 | snv | 51 |
| 28 | *SMC1A* | C>C/T | X | 53432008 | snv | 91 |
| 83 | *SMC1A* | C>C/T | X | 53432008 | snv | 95 |
| 86 | *SMC1A* | T>T/C | X | 53430710 | snv | 93 |
| 121 | *SMC3* | A>A/C | 10 | 112362759 | snv | 47 |
| 1 | *SRSF2* | G>G/T | 17 | 74732959 | snv | 33 |
| 6 | *SRSF2* | G>G/T | 17 | 74732959 | snv | 41 |
| 10 | *SRSF2* | G>G/T | 17 | 74732959 | snv | 41 |
| 13 | *SRSF2* | G>G/A | 17 | 74732959 | snv | 41 |
| 27 | *SRSF2* | G>G/C | 17 | 74732959 | snv | 44 |
| 28 | *SRSF2* | G>G/C | 17 | 74732959 | snv | 45 |
| 33 | *SRSF2* | G>G/C | 17 | 74732959 | snv | 43 |
| 34 | *SRSF2* | G>G/T | 17 | 74732959 | snv | 49 |
| 35 | *SRSF2* | G>G/GGGC | 17 | 74732959 | insertion | 37 |
| 38 | *SRSF2* | G>G/C | 17 | 74732959 | snv | 51 |
| 41 | *SRSF2* | G>G/A | 17 | 74732959 | snv | 50 |
| 45 | *SRSF2* | G>G/C | 17 | 74732959 | snv | 49 |
| 48 | *SRSF2* | G>G/T | 17 | 74732959 | snv | 47 |
| 61 | *SRSF2* | G>G/T | 17 | 74732959 | snv | 40 |
| 63 | *SRSF2* | G>G/A | 17 | 74732959 | snv | 50 |
| 64 | *SRSF2* | G>G/A | 17 | 74732959 | snv | 51 |
| 83 | *SRSF2* | G>G/T | 17 | 74732959 | snv | 43 |
| 111 | *SRSF2* | G>G/A | 17 | 74732959 | snv | 41 |
| 115 | *SRSF2* | G>G/T | 17 | 74732959 | snv | 21 |
| 123 | *SRSF2* | G>G/T | 17 | 74732959 | snv | 19 |
| 34 | *STAG2* | C>C/T | X | 123179197 | snv | 4 |
| 36 | *SUZ12* | G>G/A | 17 | 30264461 | snv | 48 |
| 16 | *TERT* | A>A/C | 5 | 1294186 | snv | 49 |
| 86 | *TERT* | G>G/A | 5 | 1293767 | snv | 53 |
| 1 | *TET2* | A>A/AT | 4 | 106157402 | insertion | 36 |
| 6 | *TET2* | C>C/T | 4 | 106156729 | snv | 46 |
| 8 | *TET2* | CA>CA/C | 4 | 106156480 | deletion | 44 |
| 8 | *TET2* | G>G/A | 4 | 106190855 | snv | 45 |
| 10 | *TET2* | C>C/T | 4 | 106164778 | snv | 44 |
| 13 | *TET2* | TC>TC/T | 4 | 106193979 | deletion | 39 |
| 13 | *TET2* | CTT>CTT/C | 4 | 106197177 | deletion | 33 |
| 16 | *TET2* | AC>AC/A | 4 | 106155513 | deletion | 43 |
| 16 | *TET2* | C>C/T | 4 | 106190860 | snv | 42 |
| 17 | *TET2* | C>C/G | 4 | 106180792 | snv | 44 |
| 18 | *TET2* | T>T/C | 4 | 106164767 | snv | 93 |
| 21 | *TET2* | GAAAGGT>GAAAGGT/G | 4 | 106162582 | deletion | 42 |
| 21 | *TET2* | G>G/A | 4 | 106162586 | snv | 82 |
| 25 | *TET2* | G>G/C | 4 | 106180838 | snv | 42 |
| 26 | *TET2* | AAT>AAT/A | 4 | 106158479 | deletion | 44 |
| 26 | *TET2* | CTAT>CTAT/C | 4 | 106164071 | deletion | 40 |
| 26 | *TET2* | GC>GC/G | 4 | 106164076 | deletion | 41 |
| 27 | *TET2* | GATTGA>GATTGA/G | 4 | 106156791 | deletion | 41 |
| 29 | *TET2* | C>C/G | 4 | 106193794 | snv | 16 |
| 29 | *TET2* | T>T/C | 4 | 106197285 | snv | 54 |
| 30 | *TET2* | TC>TC/T | 4 | 106155748 | deletion | 41 |
| 30 | *TET2* | A>A/G | 4 | 106182914 | snv | 44 |
| 33 | *TET2* | T>T/TA | 4 | 106155673 | insertion | 41 |
| 33 | *TET2* | C>C/A | 4 | 106156862 | snv | 41 |
| 34 | *TET2* | C>C/CA | 4 | 106157873 | insertion | 43 |
| 35 | *TET2* | T>T/TGC | 4 | 106155729 | insertion | 42 |
| 35 | *TET2* | C>C/T | 4 | 106193748 | snv | 42 |
| 36 | *TET2* | C>C/CT | 4 | 106158442 | insertion | 43 |
| 36 | *TET2* | C>C/CAG | 4 | 106196261 | insertion | 39 |
| 37 | *TET2* | AC>AC/A | 4 | 106182952 | deletion | 43 |
| 39 | *TET2* | T>T/TA | 4 | 106182970 | insertion | 45 |
| 39 | *TET2* | AG>AG/A | 4 | 106196772 | deletion | 44 |
| 41 | *TET2* | C>C/T | 4 | 106156072 | snv | 98 |
| 43 | *TET2* | C>C/T | 4 | 106157845 | snv | 94 |
| 45 | *TET2* | T>T/A | 4 | 106196829 | snv | 54 |
| 46 | *TET2* | CT>CT/C | 4 | 106157205 | deletion | 44 |
| 46 | *TET2* | T>T/TA | 4 | 106157794 | insertion | 45 |
| 48 | *TET2* | AACTT>AACTT/A | 4 | 106157177 | deletion | 39 |
| 50 | *TET2* | TC>TC/T | 4 | 106155920 | deletion | 45 |
| 53 | *TET2* | C>C/G | 4 | 106158275 | snv | 47 |
| 53 | *TET2* | A>A/G | 4 | 106190861 | snv | 49 |
| 61 | *TET2* | G>G/GA | 4 | 106155340 | insertion | 44 |
| 61 | *TET2* | A>A/G | 4 | 106183004 | snv | 39 |
| 62 | *TET2* | C>C/T | 4 | 106157074 | snv | 47 |
| 63 | *TET2* | T>T/C | 4 | 106164758 | snv | 97 |
| 82 | *TET2* | T>T/TG | 4 | 106196492 | insertion | 41 |
| 82 | *TET2* | G>G/GA | 4 | 106196718 | insertion | 44 |
| 83 | *TET2* | C>C/T | 4 | 106157002 | snv | 46 |
| 84 | *TET2* | A>A/AT | 4 | 106164929 | insertion | 44 |
| 84 | *TET2* | T>T/G | 4 | 106190851 | snv | 49 |
| 111 | *TET2* | GGC>GGC/G | 4 | 106157960 | deletion | 43 |
| 111 | *TET2* | G>G/A | 4 | 106162495 | snv | 45 |
| 114 | *TET2* | TCCCCAGTGTTGAAACAGCA>TCCCCAGTGTTGAAACAGCA/T | 4 | 106157185 | deletion | 14 |
| 114 | *TET2* | C>C/A | 4 | 106197318 | snv | 10 |
| 114 | *TET2* | G>G/T | 4 | 106197405 | snv | 23 |
| 115 | *TET2* | TC>TC/T | 4 | 106155920 | deletion | 35 |
| 115 | *TET2* | C>C/T | 4 | 106157059 | snv | 38 |
| 117 | *TET2* | C>C/T | 4 | 106156747 | snv | 31 |
| 121 | *TET2* | C>C/T | 4 | 106157845 | snv | 38 |
| 121 | *TET2* | G>G/C | 4 | 106180784 | snv | 42 |
| 122 | *TET2* | CT>CT/C | 4 | 106190850 | deletion | 41 |
| 122 | *TET2* | CT>CT/C | 4 | 106197180 | deletion | 41 |
| 123 | *TET2* | T>T/C | 4 | 106197285 | snv | 85 |
| 48 | *TIMM50* | TG>TG/T | 19 | 39971376 | deletion | 38 |
| 25 | *U2AF1* | G>G/A | 21 | 44524456 | snv | 43 |
| 32 | *U2AF1* | T>T/C | 21 | 44514777 | snv | 43 |
| 114 | *U2AF1* | T>T/TCTCATA | 21 | 44514769 | insertion | 13 |
| 3 | *UMODL1* | A>A/G | 21 | 43539257 | snv | 46 |
| 4 | *UMODL1* | G>G/A | 21 | 43531190 | snv | 50 |
| 33 | *UMODL1* | G>G/C | 21 | 43543122 | snv | 52 |
| 121 | *UMODL1* | G>G/A | 21 | 43491492 | snv | 48 |
| 32 | *UMODL1,C21orf128* | G>G/A | 21 | 43523985 | snv | 51 |
| 17 | *ZRSR2* | G>G/A | X | 15840875 | snv | 97 |
| 19 | *ZRSR2* | G>G/C | X | 15841228 | snv | 6 |
| 19 | *ZRSR2* | C>C/CAGCCGG | X | 15841230 | insertion | 19 |
| 29 | *ZRSR2* | C>C/T | X | 15833879 | snv | 28 |
| 30 | *ZRSR2* | C>C/T | X | 15822297 | snv | 83 |
| 31 | *ZRSR2* | GGA>GGA/G | X | 15841008 | deletion | 38 |
| 49 | *ZRSR2* | G>G/C | X | 15841228 | snv | 13 |
| 49 | *ZRSR2* | C>C/CAGCCGG | X | 15841230 | insertion | 39 |
| 82 | *ZRSR2* | G>G/A | X | 15822321 | snv | 11 |
| 117 | *ZRSR2* | AAGAG>AAGAG/A | X | 15822271 | deletion | 67 |
| 122 | *ZRSR2* | C>C/T | X | 15809121 | snv | 83 |

chr: chromosome; VAF: variant allele frequency; snv: single nucleotide variant

**Supplementary Table 2**. List of all the affected genes and frequency in the cohort of CMML patients in samples at diagnosis (n=56).

| **Gene** | **Number of patients** | **Cohort frequency** | **Gene** | **Number of patients** | **Cohort frequency** |
| --- | --- | --- | --- | --- | --- |
| *TET2* | 40 | 71% | *PHF6* | 2 | 4% |
| *ASXL1* | 24 | 43% | *SFPQ* | 2 | 4% |
| *SRSF2* | 20 | 36% | *TERT* | 2 | 4% |
| *RUNX1* | 13 | 23% | *MECOM* | 1 | 2% |
| *ZRSR2* | 9 | 16% | *ATRX* | 1 | 2% |
| *CBL* | 7 | 13% | *BCOR* | 1 | 2% |
| *NRAS* | 7 | 13% | *CDH3* | 1 | 2% |
| *EZH2* | 5 | 9% | *BRAF* | 1 | 2% |
| *CREBBP* | 5 | 9% | *CUX1* | 1 | 2% |
| *UMODL1* | 5 | 9% | *EP300* | 1 | 2% |
| *SETBP1* | 5 | 9% | *FLT3* | 1 | 2% |
| *SH2B3* | 5 | 9% | *GATA1* | 1 | 2% |
| *NF1* | 4 | 7% | *GCAT* | 1 | 2% |
| *IDH2* | 4 | 7% | *GATA2* | 1 | 2% |
| *SF3B1* | 4 | 7% | *IDH1* | 1 | 2% |
| *KMT2D* | 4 | 7% | *KIT* | 1 | 2% |
| *CSF3R* | 3 | 5% | *KMT2A* | 1 | 2% |
| *JAK2* | 3 | 5% | *PDGFRA* | 1 | 2% |
| *PTPN11* | 3 | 5% | *PDGFRB* | 1 | 2% |
| *SMC1A* | 3 | 5% | *PHLPP1* | 1 | 2% |
| *U2AF1* | 3 | 5% | *RAD21* | 1 | 2% |
| *DNMT3A* | 3 | 5% | *RPS14* | 1 | 2% |
| *KRAS* | 2 | 4% | *SF1* | 1 | 2% |
| *CTNNA1* | 2 | 4% | *SF3A1* | 1 | 2% |
| *CDH13* | 2 | 4% | *SMC3* | 1 | 2% |
| *CTCF* | 2 | 4% | *STAG2* | 1 | 2% |
| *CALR* | 2 | 4% | *SUZ12* | 1 | 2% |
| *JARID2* | 2 | 4% | *TIMM50* | 1 | 2% |
| *LUC7L2* | 2 | 4% | *AEBP2* | 1 | 2% |
| *NPM1* | 2 | 4% |  |  |  |

**Supplementary Table 3**. Patients with gene mutations in regions with copy number neutral loss of heterozygosity (CNN-LOH).

| **Number of patients** | **CNN-LOH region** | **Start** | **End** | **Size** | **Mutated gene** |
| --- | --- | --- | --- | --- | --- |
| 4 | 4q13.3q35.2 | 70579280 | 190921709 | *120342429* | *TET2* |
|  | 4q13.3q35.2 | 89992346 | 190921709 | 100.929.363 |  |
|  | 4q13.3qter | 111.229.207 | 79.692.502 | 190.921.709 |  |
|  | 4q13.3qter | 108.964.973 | 81.956.736 | 190.921.709 |  |
| 3 | 11q13.3q25 | 65018466 | 134942626 | 69.924.160 | *CBL* |
|  | 11q13.2q25 | 67.393.850 | 134938470 | 67.544.620 |  |
|  | 11q13.2q25 | *70339930* | 134939692 | *64599762* |  |
| 1 | 7q22.1q36.3 | 98915957 | 159119220 | 60.203.263 | *EZH2* |
| 1 | 17q25.3 | 78.966.914 | 81.041.938 | 2.075.024 | *SRSF2* |
| 1 | 12q21.2q24.33 | 75.863.034 | 133777902 | 57.914.868 | *KRAS* |
| 1 | 7p12.3q21.11 | 45749533 | 77814597 | 32.065.064 | None of the studied genes |
| 1 | 10p12.1q21.1 | 29074038 | 57196819 | 28.122.781 |  |
| 1 | 13q14.11q31.3 | 43719771 | 93333822 | 49.614.051 |  |
| 1 | 14q11.2q21.3 | 20511672 | 50870199 | 30.358.527 |  |

**Supplementary Table 4**. Genes included in the 83 gene panel.

| **Gene** | **Target region (exon)** | **Gene** | **Target region (exon)** | **Gene** | **Target region (exon)** | **Gene** | **Target region (exon)** | |
| --- | --- | --- | --- | --- | --- | --- | --- | --- |
| *ABL1* | 4-9 | *EED* | full | *MECOM* | full | *SF3B1* | 10-16 |  |
| *AEBP2* | full | *EP300* | full | *KMT2A* | full | *SFPQ* | full |  |
| *ASXL1* | 9, 11, 12 | *ETV6* | full | *MLL2* | full | *SH2B3* | full |  |
| *ATRX* | full | *EZH2* | full | *MPL* | 10 | *SMC1A* | full |  |
| *BCOR* | full | *FLT3* | 14, 15, 20 | *NF1* | full | *SMC3* | full |  |
| *BCORL1* | full | *GATA1* | 2 | *NPM1* | 11, 12 | *SPARC* | full |  |
| *BRAF* | full | *GATA2* | full | *NRAS* | 1-3 | *SRSF2* | 1 |  |
| *CALR* | 9 | *GCAT* | full | *PDGFRA* | full | *STAG1* | full |  |
| *CBL* | 8, 9 | *GNAS* | full | *PDGFRB* | full | *STAG2* | full |  |
| *CBLB* | 9, 10 | *HRAS* | 2, 3 | *PHF6* | full | *SUZ12* | full |  |
| *CDH13* | full | *IDH1* | 4 | *PHLPP1* | full | *TERC* | full |  |
| *CDH3* | full | *IDH2* | 4 | *PTEN* | 5-8 | *TERT* | full |  |
| *CDKN2A* | full | *IKZF1* | full | *PTPN11* | full | *TET2* | 2-11 |  |
| *CEBPA* | full | *IRF1* | full | *RAD21* | full | *TGM2* | full |  |
| *CREBBP* | full | *JAK2* | 12-16 | *RPS14* | full | *TIMM50* | full |  |
| *CSF3R* | full | *JAK3* | 13 | *RUNX1* | 3-8 | *TP53* | 4-11 |  |
| *CSNK1A1* | full | *JARID2* | full | *SALL4* | full | *U2AF1* | 2, 6 |  |
| *CTCF* | full | *KDM6A* | full | *SBDS* | full | *UMODL1* | full |  |
| *CTNNA1* | full | *KIT* | 2,8-11,13,17 | *SETBP1* | 4 | *WT1* | 7, 9 |  |
| *CUX1* | full | *KRAS* | 1-3 | *SF1* | full | *ZRSR2* | full |  |
| *DNMT3A* | full | *LUC7L2* | full | *SF3A1* | full |  |  |  |

**Supplementary Figures**

**Supplementary Figure Legends**

**Supplementary Figure 1**. **Number and type of mutations across the CMML patients at diagnosis**. **(A)** distribution of number of mutations detected per patient; **(B)** frequency of affected genes in the entire cohort; **(C)** type of mutation; **(D)** mechanisms in which the main affected genes are involved.

**Supplementary Figure 2**. **Prognostic impact of gene mutations**. **(A)** OS and PFS curves according to number of total mutations; **(B)** OS and PFS curves according to mutations in individual genes (*ASXL1*, *EZH2, NRAS, SRSF2* and *TET2*); **(C)** OS and PFS curves according to number of adverse risk gene mutations (*ASXL1*, *EZH2, NRAS*, *SRSF2*); **(D)** OS and PFS curves according to combinations between *ASXL1* and *TET2* mutations. See Table 3 for 3-year percentage overall survival and progression free survival and confidence intervals. AR mutations: adverse risk gene mutations (*ASXL1*, *EZH2*, *NRAS, SRSF2*). **(E)** OS and PFS curves according to combinations between *TET2* mutations and adverse risk genes (excluding *ASXL1*). See Table 3 for 3-year percentage overall survival and progression free survival and confidence intervals. AR mutations excluding *ASXL1* (*EZH2*, *NRAS, SRSF2*).

**Supplementary Figure 1**

**
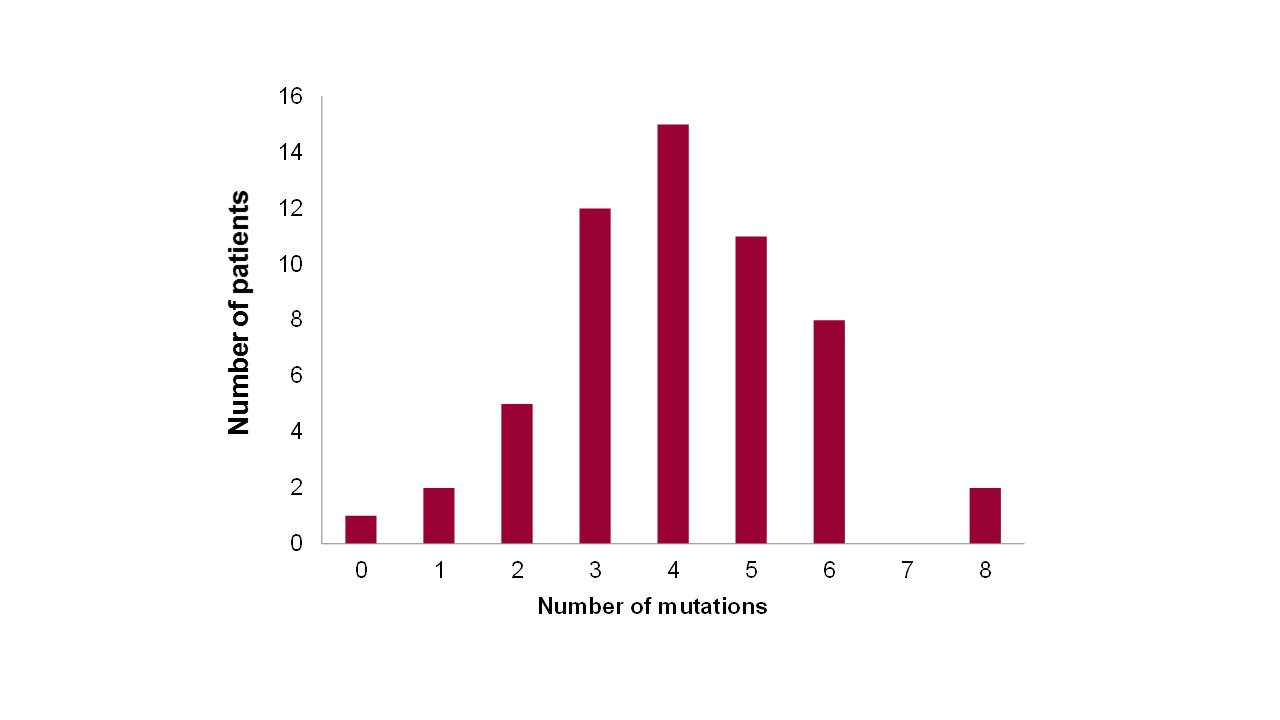
A**

**
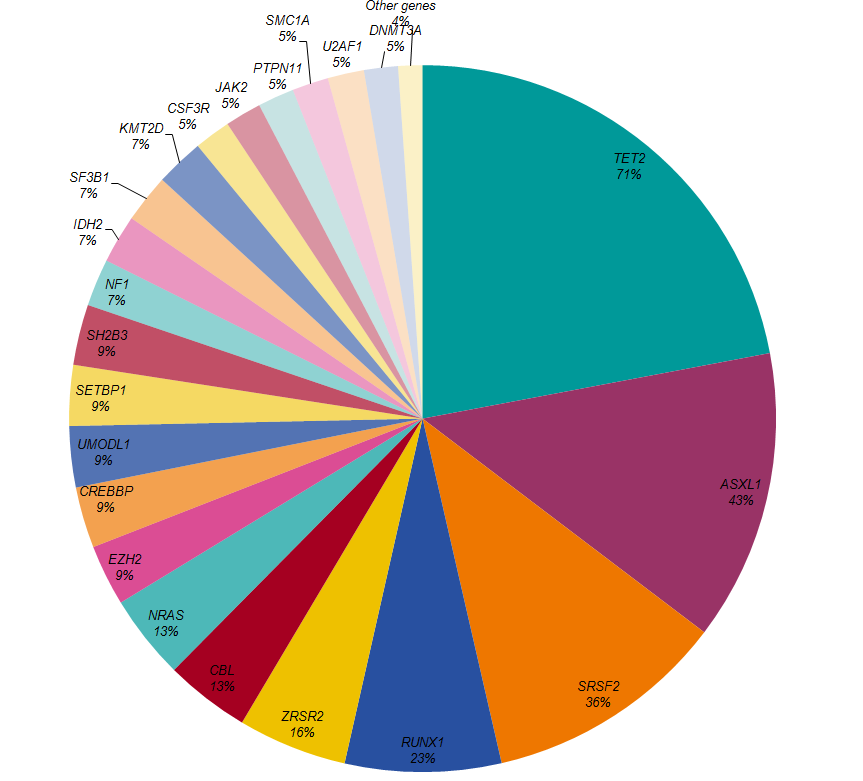
**

**B**

**C**

**
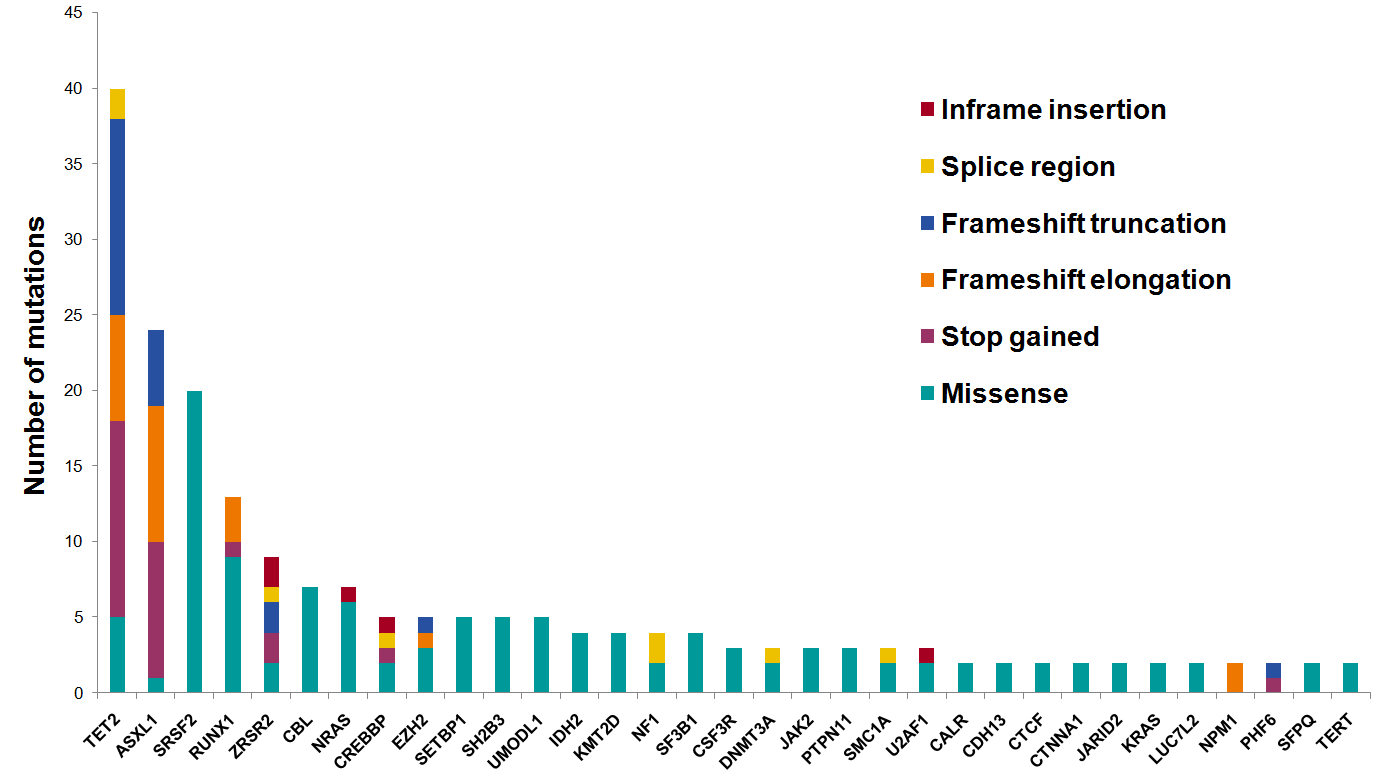
**

**
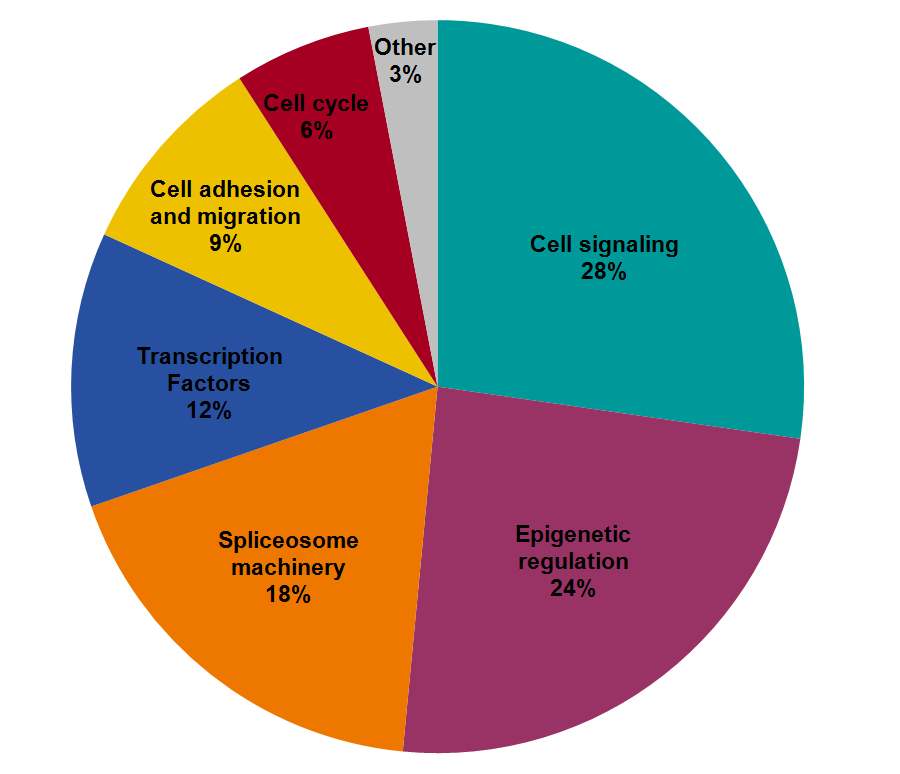
D**

**Supplementary Figure 2**

**A**


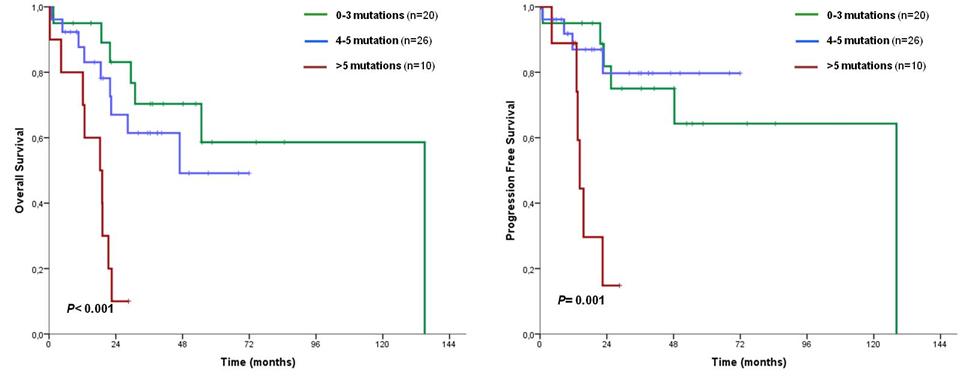


**B**


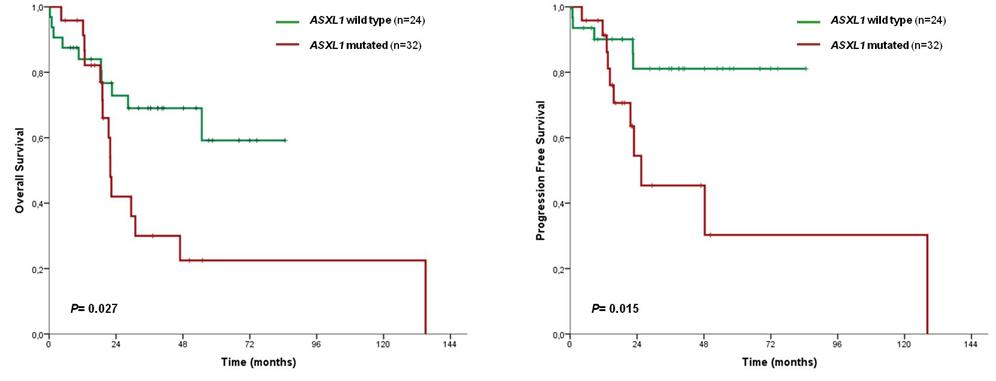


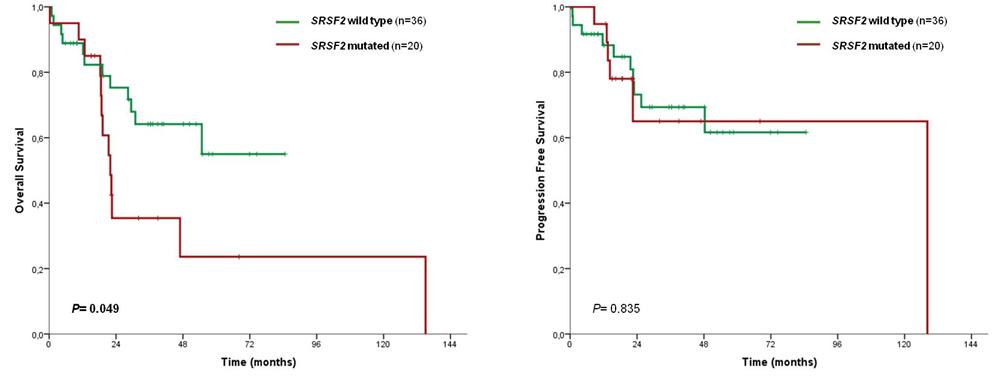


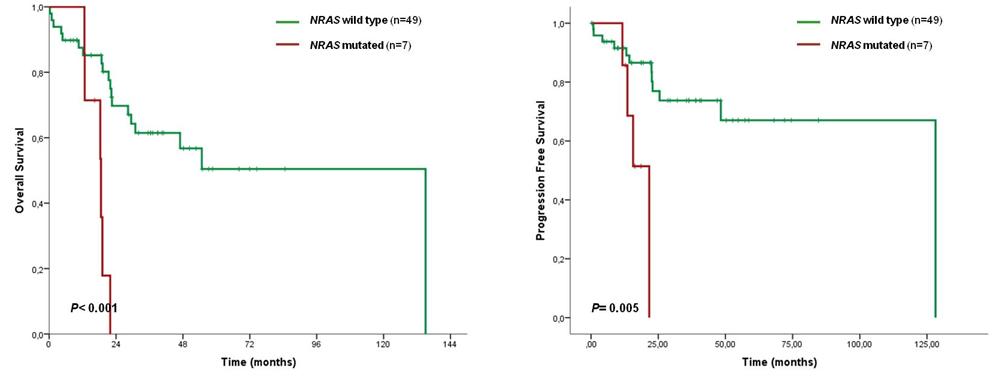


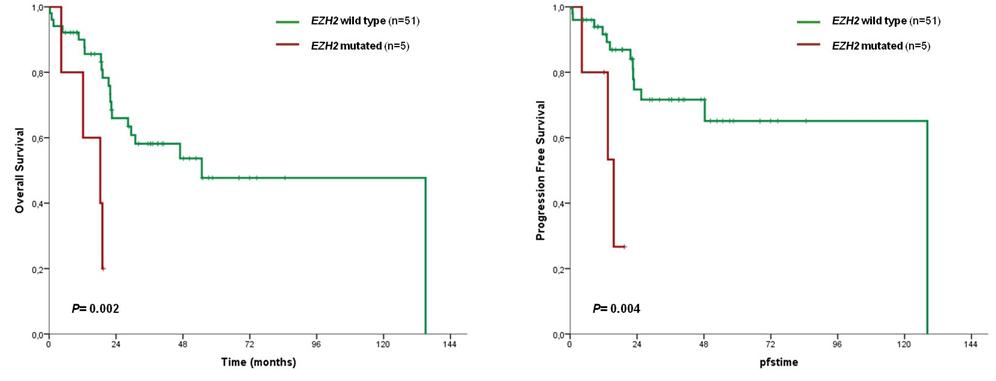


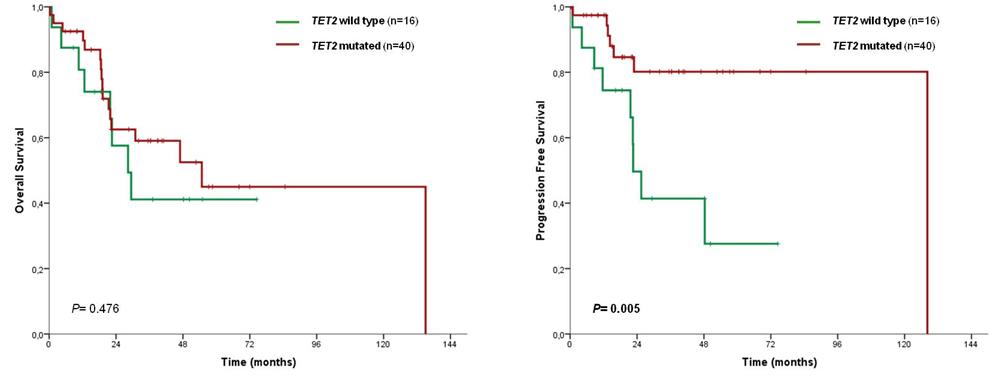


**C**


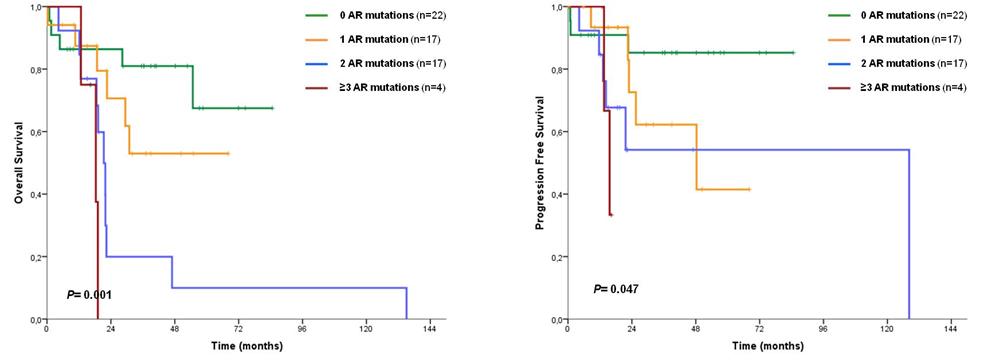


**D**


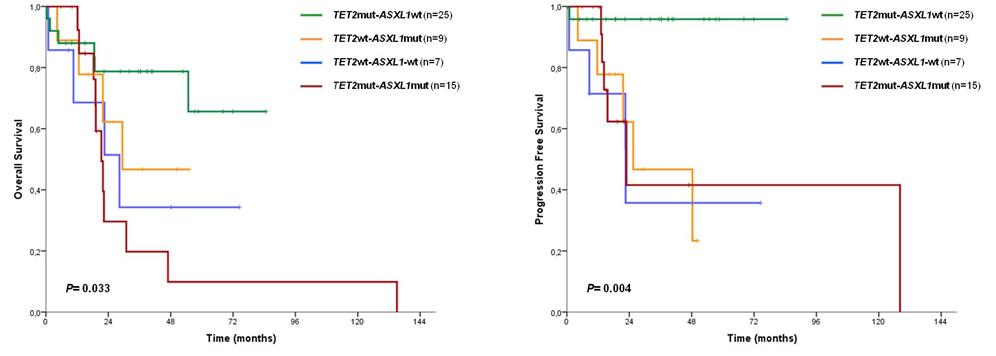


**E**


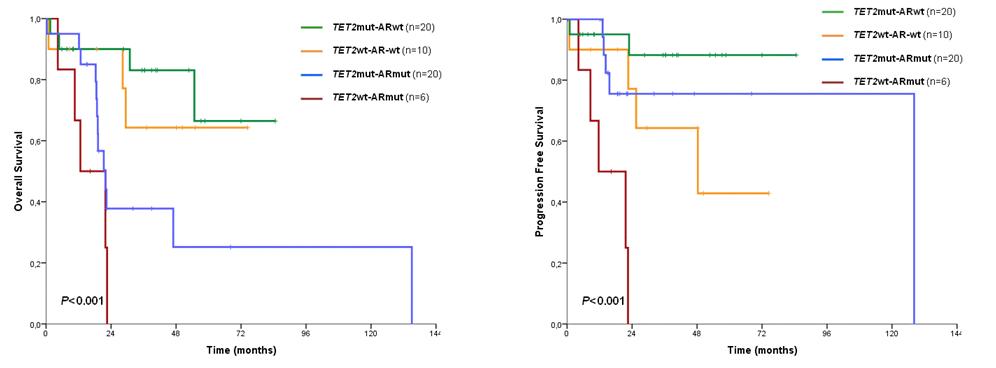

Supplement: Supplementary file 2 [file oncotarget-07-57021-s002.docx]
